# Review of Si-Based GeSn CVD Growth and Optoelectronic Applications

**DOI:** 10.3390/nano11102556

**Published:** 2021-09-29

**Authors:** Yuanhao Miao, Guilei Wang, Zhenzhen Kong, Buqing Xu, Xuewei Zhao, Xue Luo, Hongxiao Lin, Yan Dong, Bin Lu, Linpeng Dong, Jiuren Zhou, Jinbiao Liu, Henry H. Radamson

**Affiliations:** 1 Key Laboratory of Microelectronic Devices Integrated Technology, Institute of Microelectronics, Chinese Academy of Sciences, Beijing 100029, China; wangguilei@ime.ac.cn (G.W.); kongzhenzhen@ime.ac.cn (Z.K.); xubuqing@ime.ac.cn (B.X.); zhaoxuewei@ime.ac.cn (X.Z.); dongyan2019@ime.ac.cn (Y.D.); liujinbiao@ime.ac.cn (J.L.); 2 Research and Development Center of Optoelectronic Hybrid IC, Guangdong Greater Bay Area Institute of Integrated Circuit and System, Guangzhou 510535, China; luoxue@giics.com.cn (X.L.); linhongxiao@giics.com.cn (H.L.); lubinsxnu@sina.cn (B.L.); lpdong@xatu.edu.cn (L.D.); 3 Institute of Microelectronics, University of Chinese Academy of Sciences, Beijing 100049, China; 4 School of Physics and Information Engineering, Shanxi Normal University, Linfen 041004, China; 5 Shaanxi Province Key Laboratory of Thin Films Technology Optical Test, Xi’an Technological University, Xi’an 710032, China; 6 Department of Electrical and Computer Engineering, National University of Singapore, Singapore 117576, Singapore; zhoujiuren@163.com

**Keywords:** GeSn, CVD, lasers, detectors, transistors

## Abstract

GeSn alloys have already attracted extensive attention due to their excellent properties and wide-ranging electronic and optoelectronic applications. Both theoretical and experimental results have shown that direct bandgap GeSn alloys are preferable for Si-based, high-efficiency light source applications. For the abovementioned purposes, molecular beam epitaxy (MBE), physical vapour deposition (PVD), and chemical vapor deposition (CVD) technologies have been extensively explored to grow high-quality GeSn alloys. However, CVD is the dominant growth method in the industry, and it is therefore more easily transferred. This review is focused on the recent progress in GeSn CVD growth (including ion implantation, in situ doping technology, and ohmic contacts), GeSn detectors, GeSn lasers, and GeSn transistors. These review results will provide huge advancements for the research and development of high-performance electronic and optoelectronic devices.

## 1. Introduction

Si-based integrated circuits (ICs), which are dominated by Si CMOS technology, have reached their physics limit. The influences of quantum effects, parasitic parameters, and process parameters on data transmission applications are also reaching their limits, as the rapid development of microelectronics has led to higher requirements for data transmission technology. For these reasons, scientists have proposed schemes to integrate optoelectronic devices with microelectronic devices [1,2,3,4,5,6,7]. However, Si-based on-chip integrated light source was lacking, and the light sources for existing optoelectronic integrated circuits (OEICs) were all externally coupled; though the coupling efficiency between the edge of the light source and grating coupler was high enough, the lack of an on-chip light source restricted OEICs’ applications [8,9,10]. As such, many research programs started to pay more attention to Si-based monolithic OEIC technology [11,12,13,14,15], which has the following advantages over the baseline technology: (i) it is compatible with mature Si CMOS technology; (ii) has low costs; (iii) has larger wafer sizes and larger scale production; (iv) its partial electrical interconnection can be replaced by optical interconnection, which can realize high-efficiency, high-speed, and low loss data transmission. Si-based monolithic OEIC technology uses Si-compatible semiconductor technology to integrate optoelectronic devices into Si chips in order to improve chip performance, extend chip function, and reduce costs. Though Si-based photonic devices, such as optical waveguides [16,17], photodetectors [18,19,20], optical modulators [21,22,23], and optical switches [24,25], have been successfully developed, it is difficult to achieve high-efficiency emission due to the facts that Si is an indirect bandgap semiconductor and its light emission efficiency is about five orders of magnitude lower than that of direct band gap III–V compound semiconductors. Thus, the need for an Si-based high-efficiency light source represents an important technical bottleneck in the development of Si-based monolithic OEICs. Therefore, looking for a direct bandgap semiconductor material that is compatible with the Si CMOS process is of great significance in the creation of large scale Si-based monolithic OEICs [26,27,28].

Group IV materials are compatible with the traditional Si CMOS process, and Si, SiGe, and Ge are commonly used as indirect band gap semiconductors despite not being suitable for light emission. Fortunately, tensile strain engineering and Sn-alloying engineering have enabled Ge to become a quasi-direct bandgap or direct bandgap material due to the small bandgap difference between its two minima in conduction bands (only 136 meV). Experimental research has shown an optical gain of 0.24% for tensile-strained n^+^-type Ge (the n-type doping level is 1 × 10^19^ cm^−3^), which led to the creation of optically injected and electrically injected Ge lasers [29,30,31,32]. However, the threshold for a Ge laser is too high, which means that weak tensile-strained n^+^-type Ge is not able to supply enough optical gain to achieve low-threshold lasing.

In recent decades, GeSn alloys have demonstrated novel indirect-to-direct bandgap transition, as well excellent carrier transport. Due to their tunable band structures, GeSn materials have become promising candidates to create Si-based OEICs with higher hole mobility, enhanced light absorption, etc. [33,34,35,36,37]. Growing high-quality GeSn layers with relatively high Sn contents has different challenges, e.g., Sn segregation during growth and the poor thermal stability of SnGe layers [38,39,40,41]. These issues root from the low solid solubility of Sn in Ge (<1%) and the large lattice mismatch between Si or Ge and GeSn. As early as 1995, the first growth of a GeSn/Ge superlattice was reported using a very low growth temperature in a molecular beam epitaxy (MBE) chamber. Such GeSn layers had an Sn content of 26% [42,43]. Based on these early pioneer works, other growth techniques, such as chemical vapor deposition (CVD) and magnetron sputtering, have been widely used to grow high-quality direct bandgap GeSn materials with high Sn contents [44,45,46,47,48,49]. Although MBE can grow GeSn materials well, its growth rate is extremely low, which makes it tough to manufacture on a large scale. To achieve a significant impact within the industry, it is very important to develop a commercially available tool to grow high-quality GeSn materials. At a very early development stage of GeSn growth via CVD, SnD_4_ and Ge_2_H_6_ were chosen as the Sn precursors and Ge precursors, respectively. Although there were many foundational studies on GeSn growth via CVD, SnD_4_ is a high-cost material with a short lifetime, which makes it incompatible with the industry. For this reason, other precursors such as SnCl_4_ have been explored. The IMEC and KTH groups pioneered the growing of GeSn layers using commercially available reaction precursors (SnCl_4_/Ge_2_H_6_) [50]. A major breakthrough was later demonstrated using the production of commercially available reaction precursors (SnCl_4_/GeH_4_) [51,52]. The limitations of incorporating Sn into Ge have been conquered, and two major breakthroughs for GeSn CVD growth have been reached: (i) a world record high Sn content (22.3%) in bulk GeSn materials with PL emission was observed at room temperature (indicating good material quality), and (ii) SiGeSn/GeSn/SiGeSn multiple quantum well (MQW) structure growth and low-temperature PL intensity were later able to be remarkable enhanced [53,54,55,56]. Furthermore, the low costs and widespread availability of these chemicals in large-scale fabrication makes them the best choice for GeSn-based optoelectronic integration into CMOS processing. To make GeSn an efficient N-type or P-type semiconductor material for optoelectronic device application, there is an urgent need to research and develop doping engineering for GeSn. Currently, doping technologies, such as ion implantation and in situ CVD doping, have been optimized regarding their target doping concentration and doping distributions.

After the successful growth of P^+^-Si/i–GeSn/n–GeSn via CVD, Jay Mathews et al. demonstrated the world’s first GeSn photodetector with a 2% Sn content in 2009 [57]. The wavelength cutoff was extended to be at least 1750 nm, which means that the GeSn photodetector with a 2% Sn content can cover the entire telecommunication band. Since then, GeSn photoconductor detectors [58,59,60,61,62,63], and p–GeSn/i–GeSn/n–GeSn heterostructure detectors [64,65,66,67,68] have been demonstrated. Advances in GeSn CVD growth technology have occurred alongside material quality and detector performance improvements, including: (i) the wavelength cutoff for the GeSn photodetector has been progressively broadened from 1800 nm to 2100, 2400, 2600, 2650, and 3650 nm [63]; (ii) based on wafer-bonding technology, the dark current for GeSn photodetector has been suppressed by more than two orders of magnitude [69]; (iii) peak specific detectivity values are now comparable to those of commercial extended-InGaAs detectors (4 × 10^10^ cm·Hz^1/2^·W^−1^) at the same wavelength range; (iv) a passivation technique was developed to enhance responsivity and peak specific detectivity [65]; and (v) mid-IR imaging was demonstrated with GeSn photodetectors, and the image quality of the GeSn photodetectors was found to be superior to that of a commercial PbSe detector [63].

Alongside the significant breakthroughs in GeSn growth and detectors, GeSn lasing had also developed to an advanced stage. Recently reported GeSn laser structures have all been grown via the CVD technique. Following the observation of a PL peak with narrowed line widths, a true direct bandgap GeSn material with an Sn content of up to 10% was experimentally demonstrated in 2014 [33]. Encouraged by this major technical breakthrough, researchers used the injection methods such as optical injection with a Ge laser to check the GeSn waveguide, and lasing behavior was clearly observed at a low temperature in 2015 [70]. Following this breakthrough, several types of GeSn lasers [71,72,73,74,75,76,77,78,79,80,81,82] were demonstrated, though they still suffer from the problems of low-temperature operation and high lasing thresholds. To overcome these difficulties, several methods have been proposed to improve performance, such as greater Sn incorporation into Ge [73,75,76], the use of SiGeSn/GeSn/SiGeSn heterostructures or SiGeSn/GeSn/SiGeSn MQWs as the gain medium [83,84,85,86], a modulation doping scheme in SiGeSn/GeSn/SiGeSn MQWs [87], defect management [80], and thermal management [81,82]. Considerable efforts in GeSn lasing research have led to an increased maximum lasing temperature of 270 K [76] due to the amazing discovery of strain relaxation growth mechanism [88]. Near-room-temperature lasing was also observed for a GeSn active medium with a 16% Sn content and high uniaxial tensile strain [77]. A breakthrough regarding the optical pumping threshold was reported in 2020, when a low-Sn-content GeSn material with a high uniaxial tensile strain was utilized as an active medium; continuous wave (CW) lasing was also achieved. However, the lasing temperature only reached 100 K due to the low directness of the active medium [80]. In the same year, electrically pumped GeSn/SiGeSn heterostructure lasers with operation temperatures of up to 100 K were demonstrated [89,90]; this was an essential achievement for Si-based electrically pumped group IV interband lasing.

As a group IV material, GeSn is compatible with Si and can realize the transition from indirect band gap to direct band gap by adjusting its Sn content, which makes it the best substitute for group IV materials in Si-based optoelectronic integration applications. GeSn has an extremely high carrier mobility, so it may also be an ideal materials for transistor applications. Due to the significant development of GeSn CVD growth technology, vertically stacked 3-GeSn-nanosheet pGAAFETs (gate-all-around FETs) [91], GeSn p-FinFETs [92,93], GeSn n-channel MOSFETs [94,95], GeSn/Ge vertical nanowire pFETs [96], GeSn GAA nanowire pFETs [97], and GeSn n-FinFETs [98] have been successfully demonstrated. Additionally, GeSn’s direct band gap property was found to effectively improve the tunneling probability of electrons, making an excellent material for TFET preparation [99,100], this opening a new development direction for the integrated circuit after Moore’s era. The discovery of this property has attracted considerable research interest in recent years. Since Sn naturally has low solid solubility in Ge (smaller than 1%), growth of high Sn composition single crystal GeSn is difficult. At present, devices prepared with GeSn materials are still in the research and development stage, so they have not been widely used in production.

To the best of our knowledge, there has yet to be a review article that systematically reported on GeSn material growth and counterpart optoelectronic devices using the CVD technique. UHVCVD [101,102,103,104], RPCVD [105,106,107,108,109,110], PECVD [111,112,113], LPCVD [114,115,116,117], and APCVD [118,119] are discussed in this review, with a focus on identifying processes that can be transferred for the commercial production of GeSn. The objective of this comprehensive review article is to provide readers with a full understanding of the recent experimental advancements in GeSn material growth using CVD, as well as their optoelectronic applications. However, due to the large numbers of publications in this area, the authors of this work only selected articles with significant scientific impacts.

## 2. Research Progress for GeSn CVD Growth and Its Potential Applications

So far, several types of growth techniques, such as MBE, magnetron sputtering, and CVD have been used to grow GeSn materials. CVD is the dominant growth method in the industry, so more easily transferable. Therefore, we decided to review GeSn CVD growth and its potential applications.

### 2.1. Potential Applications

**Figure 1:**
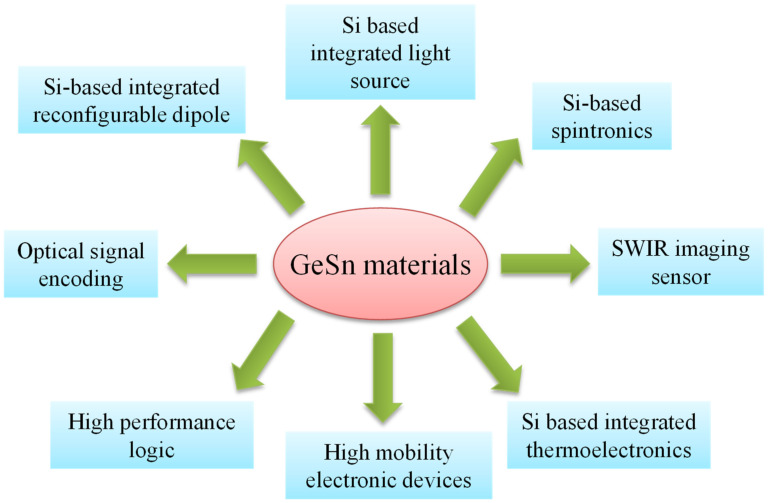
Potential applications of GeSn materials in different research areas.

**Figure 2:**
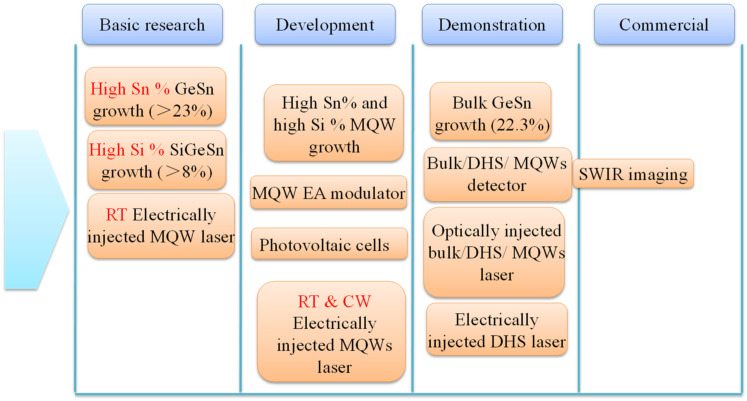
Optoelectronic applications of GeSn as a function of technology readiness level (GeSn transistors, which are still in the technical development stage, are not shown here due to space limitations).

A literature survey revealed that GeSn materials have numerous potential applications, including Si-based, integrated, high-efficiency light sources [120,121,122]; high-mobility electronic devices [92,93,94,95,96,97,98,99,100]; low-cost, Si-based, high-performance shortwave infrared (SWIR) imaging sensors [63,64,65]; Si-based photovoltaics [123]; optical signal encoding in the mid-infrared range [124,125]; high-performance logic applications [126,127]; Si-based integrated thermoelectrics as wearable devices [128,129]; Si-based spintronics [130,131]; Si-based integrated reconfigurable dipoles [132,133]; and Si-based quantum computing [134,135] (Figure 1). GeSn-related fundamental research and development applications have also been extensively investigated (Figure 2).

Figure 2 shows the optoelectronic applications of GeSn as a function of technology readiness level. It can be observed that GeSn detectors are getting closer to the low-cost SWIR imaging applications, indicating that GeSn materials have great potential for use in next-generation civilian night-vision and IR cameras [63,64,65]. However, there are still some technical problems, which are discussed in Section 3. In addition to detectors (which are being rapidly developed), high-quality SiGeSn/GeSn/SiGeSn MQW growth, room-temperature, CW, and electrically injected SiGeSn/GeSn/SiGeSn MQW lasers; MQW electro-absorption (EA) modulators; and photovoltaic cells are in the research and development stage.

### 2.2. Research Progress for GeSn CVD Growth

**Figure 3:**
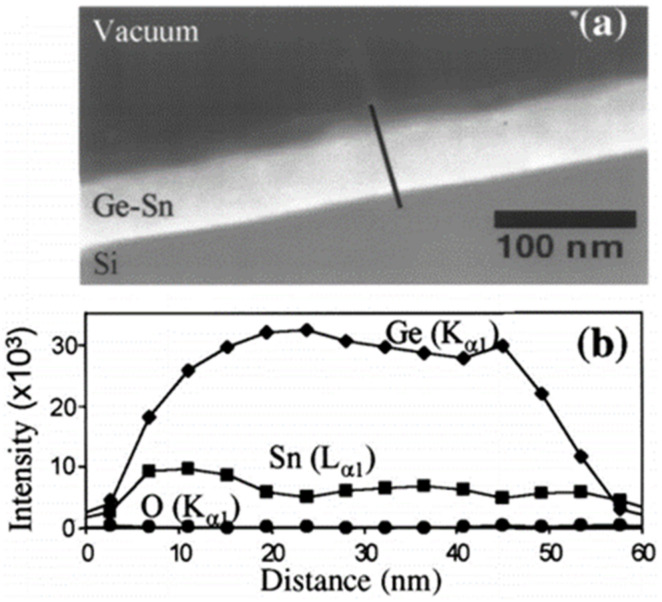
(**a**) Scanning transmission electron microscopy (STEM) image and (**b**) EDX cross-sectional profile of the GeSn with Sn contents of up to 25%. Reproduced with permission from [138], AIP Publishing, 2001.

In 2001, Kouvetakis’s group from Arizona State University (ASU) first reported a GeSn alloy on oxidized and oxidized-free Si using UHVCVD [136]; since then, extensive GeSn CVD growth-related research works have been carried out. In 2003, SnD_4_ and SiH_3_GeH_3_ were used as reaction precursors, and single-phase SiGeSn on a GeSn buffer was first achieved on Si via UHVCVD at 350 °C [137]. To create GeSn materials with higher Sn contents, SnD_4_ and Ge_2_H_6_ were chosen as Sn and Ge precursors, respectively; the experimental results showed that SnD_4_ is helpful for low-temperature growth, and its reaction with Ge_2_H_6_ can create GeSn with an Sn content of up to 25% [138] (Figure 3). The crystallinity, bandgap, lattice constants, optical properties, photoresponses, photocurrents, and Raman scattering results of GeSn materials grown by UHVCVD have been systematically demonstrated [139,140,141,142,143,144]. In order to grow GeSn at extremely low temperatures, some authors used Ge_3_H_8_ and Ge_4_H_10_ as Ge precursors [145,146]. By using this method, single crystalline GeSn alloys were successfully deposited at temperatures ranging from 300 to 330 °C, the growth rate of the allows was able to meet industrial requirements, and the traditional SK growth mode was avoided. Finally, the authors concluded that Ge_3_H_8_ is a superior solution to grow GeSn alloys via UHVCVD [145,146]. Compared with previously reported reaction precursors (SnD_4_/Ge_2_H_6_), the growth rate of the SnD_4_/Ge_3_H_8_ combination was found to be improved 3–4 times. For this reason, a 1 μm thick GeSn layer with an Sn content of up to 9% was implemented, and room temperature photoluminescence spectra were observed, indicating that GeSn has great potential to be utilized as a gain medium for a Group IV laser. Later, SiGeSn growth at ultralow temperatures (from 290 to 330 °C) using Ge_4_H_10_, Si_4_H_10_, and SnD_4_ were reported [147,148,149].

Although there have been many foundational studies on GeSn growth via CVD investigated, SnD_4_ has high costs, is incompatible with the industry, and is unstable at room temperature. For these reasons, other precursors such as SnCl_4_ have been explored. IMEC and KTH were the first groups to propose GeSn growth using commercially available reaction precursors (SnCl_4_/Ge_2_H_6_).

**Figure 4:**
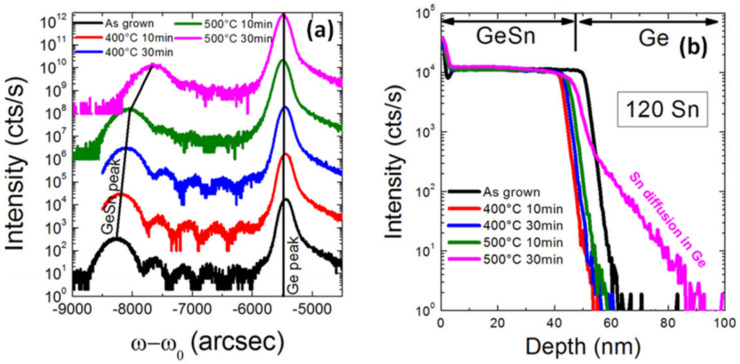
Comparison of (**a**) (004) omega-2 theta scans and (**b**) Sn content profiles of as-grown and annealed Ge_0.92_Sn_0.08_ samples under different annealing conditions. Reproduced with permission from [150], AIP Publishing, 2011.

Due to the fact that SnCl_4_ is liquid at room temperature, these groups evaporated SnCl_4_ using a bubbler that was connected to an RPCVD chamber. Experimental results showed that defect-free doped and undoped GeSn layers with Sn contents of up to 8% were created using RPCVD at atmosphere conditions. Thermal stability was further determined by annealing at different conditions (400 °C for 10 min, 400 °C for 30 min, 500 °C for 10 min, and 500 °C for 30 min); the (004) omega-2 theta scan of as-grown and annealed Ge_0.92_Sn_0.08_ samples were compared (Figure 4a). For the sample annealed at 500 °C for 30 min, the diffraction peaks of GeSn and Ge widened and a clear GeSn peak shift was observed, suggesting possible Ge–Sn interdiffusion. To further confirm this assumption, secondary ion mass spectroscopy (SIMS) was conducted. From the SIMS results, the authors concluded that APCVD-grown GeSn with 8% Sn content was stable at the annealing condition of 500 °C for 30 min (Figure 4b). This work paved the way for GeSn growth using both commercially available reaction precursors and CVD production equipment.

**Figure 5:**
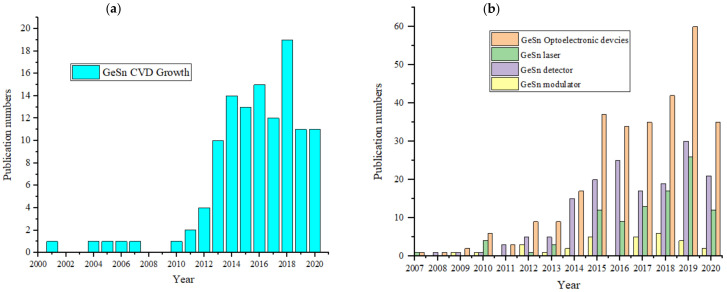
(**a**) Number of publications/year on GeSn materials grown by the CVD technique; (**b**) number of the publications/year on GeSn optoelectronic devices (theoretical calculations and conference proceedings are included).

Since then, there has been a sharp increase in the scientific knowledge of GeSn CVD growth, as shown by a number of publications (Figure 5a). The number of publications on GeSn CVD growth grew dramatically in 2013 and reaches 19 in 2018 (Figure 5a). The rapid development of GeSn CVD growth techniques has meant that the number of GeSn optoelectronic device publications followed the similar tendency (Figure 5b): (i) following the world’s first demonstration of a GeSn detector, GeSn detector-related publications grew from 1 in 2008 to 30 in 2019; (ii) since the world’s first demonstration of an optically pumped GeSn laser, publications related to GeSn lasers continually increased from 10 in 2015 to 25 in 2019, and the majority of these laser publications reported experimental results; (iii) there are still few publications regarding GeSn modulators, and a CVD-grown modulator has not been achieved (the majority of the modulator publications have been theoretical investigations).

**Figure 6:**
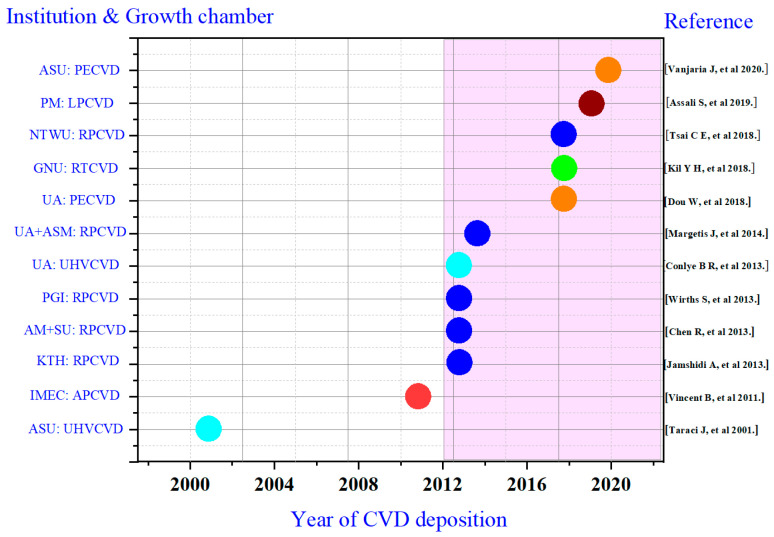
List of GeSn CVD growth papers by different groups.

To help readers to understand the research status of CVD growth techniques, Figure 6 summarizes research on GeSn CVD growth since the introduction of CVD in 2001 in terms of the research institution, growth chamber, year of deposition, and corresponding reference. Figure 6 shows several types of growth chambers, such as UHVCVD, RPCVD, APCVD, PECVD, LPCVD, and RTCVD, that have been used to grow GeSn materials. Following pioneer works from ASU and IMEC, research groups from KTH Royal Institute of Technology (KTH), Applied Materials Inc (AM), PGI (Peter Grünberg Institute), and UA (University of Arkansas) started researching GeSn growth using CVD technology in 2013. Since then, research groups from ASM, University of Warwick (UW), National Taiwan University (NTWU), and Université de Montréal (EPM) have also researched GeSn CVD growth. Among all CVD growth technologies, RPCVD growth chamber is most widely accepted due to its commercial availability and more easily transferability (six research groups have used RPCVD chambers to grow GeSn). After the successful demonstration of the low-temperature growth of high-quality Ge on Si using PECVD, plasma-enhanced techniques came to be regarded as promising methods to grow GeSn materials. Thus, plasma-enhanced GeSn growth techniques aroused researchers’ attentions from UA and ASU.

### 2.3. GeSn CVD Growth Strategy

To have a full understanding of the GeSn CVD growth strategy, it is necessary to calibrate the Ge growth at low temperatures (below 450 °C). After calibration, the flow rate between Ge precursor and Sn precursor needs to be taken into consideration due to the possible etching effect of the generated Cl* species on the GeSn surface. Therefore, there is a critical flow rate, and the growth rate for GeSn growth has to be high enough to overcome the etching rate. More importantly, the effects of temperature, pressure, carrier gas, and strain relaxation on material growth must be canvassed.

#### 2.3.1. Temperature and Pressure Effect on GeSn Growth

**Figure 7:**
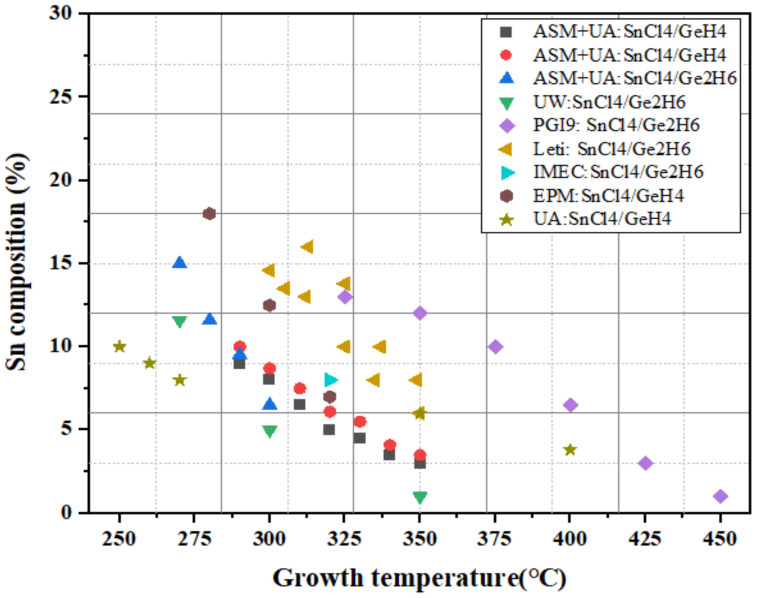
Temperature effect on the Sn content from different research groups (UW, UA, and EPM denote the University of Warwick, University of Arkansas, and Université de Montréal, respectively) [113,115,116,117,151,152,153,154,155,156].

Previous GeSn CVD growth work has demonstrated that Sn content is closely related to growth temperature because the decreasing temperature moves the growth conditions further from equilibrium, thus increasing Sn content. Therefore, we summarize most GeSn CVD growth results in Figure 7. In GeSn growth using the SnCl_4_/GeH_4_ reaction precursor combination, SnCl_4_ and GeH_4_ lose their reactivity at a temperature of 280 °C and growth is totally ceased. Below 285 °C, GeH_4_ is not well-adsorbed, which may suggest the generation of GeH_2_ and/or 2H. Therefore, the growth temperature for GeSn RPCVD growth with the SnCl_4_/GeH_4_ reaction precursor combination is usually higher than 280 °C. For the Ge_2_H_6_ and SnCl_4_ precursor combination, GeSn growth temperature could be as low as 275 °C.

**Figure 8:**
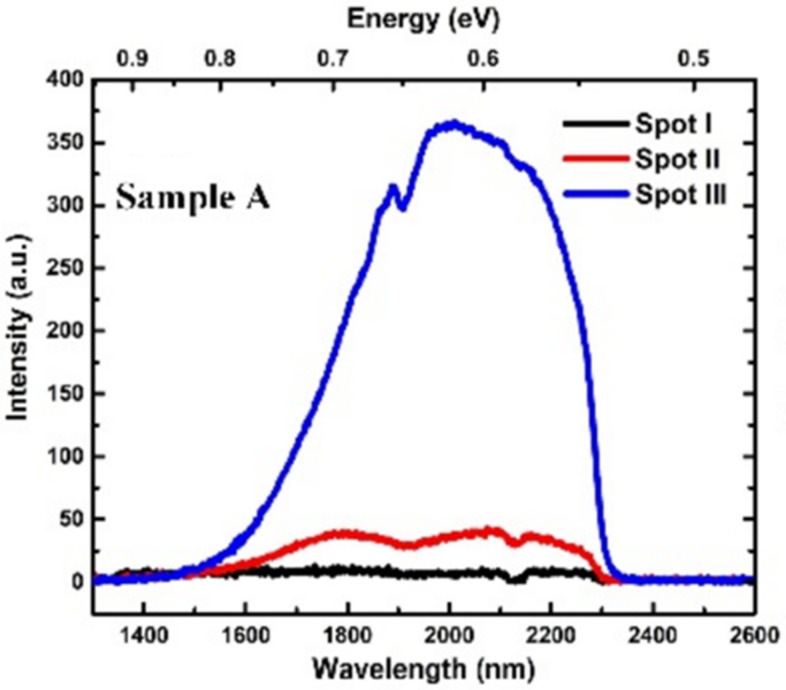
Room-temperature PL spectra for GeSn grown by PECVD technology (the Sn content is 6%). Reproduced from [114], open access by OSA Library, 2018.

Significantly, UA demonstrated GeSn growth using PECVD with the commercially available GeH_4_ and SnCl_4_; low-temperature growth at 350 °C for GeSn epitaxy on an Si substrate was achieved with an Sn content of up to 6% [113]. By using a 1064 pulsed laser as the light source, a PL signal was also observed at the peak wavelength of 2000 nm, as shown in Figure 8 (Spot III).

**Figure 9:**
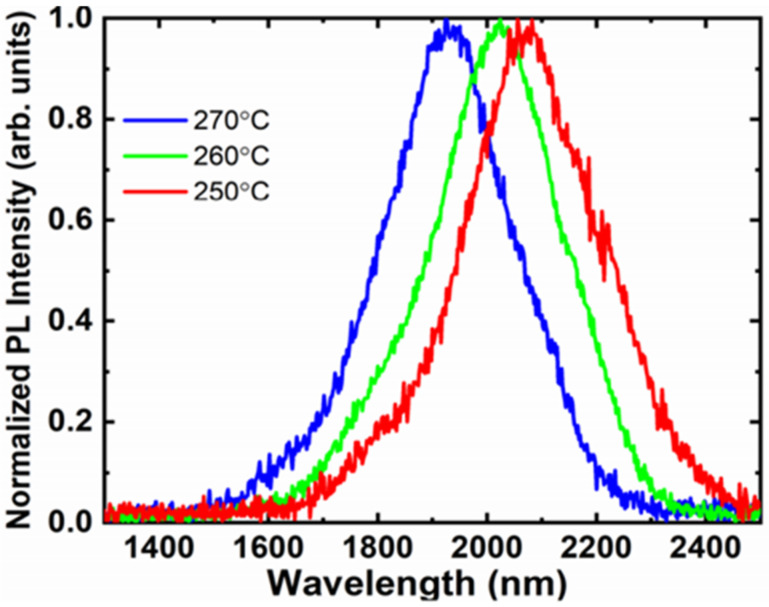
PL spectra for the GeSn grown at temperatures of 250, 260, and 270 °C. Reproduced from [157], open access by ScholarWorks@UARK.

Their follow-up work verified that the PECVD system was able to grow a high-Sn-content (>10%, with an PL emission peak at approximately 2100 nm) GeSn layer at ultralow temperatures (250, 260, and 270 °C) [157] (Figure 9). The realization of GeSn PECVD growth at such low temperatures using a SnCl_4_/GeH_4_ precursor combination mainly benefits from plasma-assisted reactivity improvements [157]. With proper growth optimization, the Sn content of the GeSn grown by PECVD should be higher than that of other CVD chambers. Compared to GeH_4_, Ge_2_H_6_ is more reactive and possesses lower growth temperature capabilities, indicating that the reactivity of the Ge-hydride is the only limiting factor for low-temperature GeSn growth. For GeSn RPCVD growth using GeH_4_, Sn incorporation was found to drastically decrease at ~285 °C, whereas the growth temperature limit for using Ge_2_H_6_ was found to be 270 °C [153].

**Figure 10:**
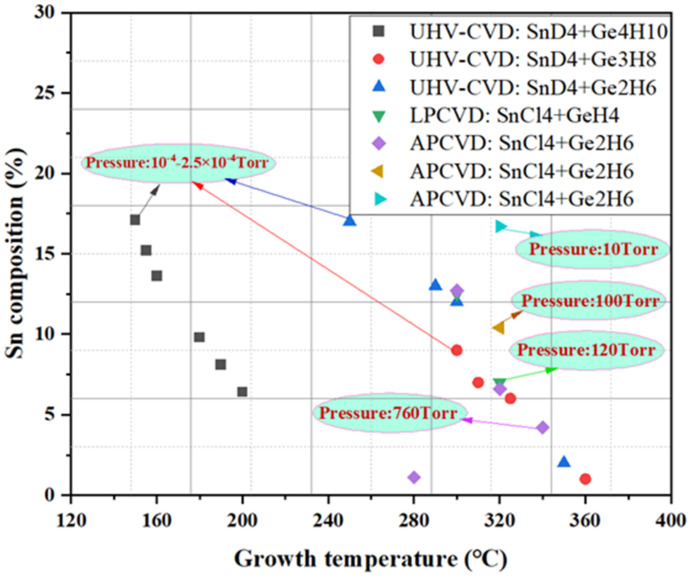
Temperature effect on Sn content from UHVCVD, LPCVD, and APCVD growth.

**Figure 11:**
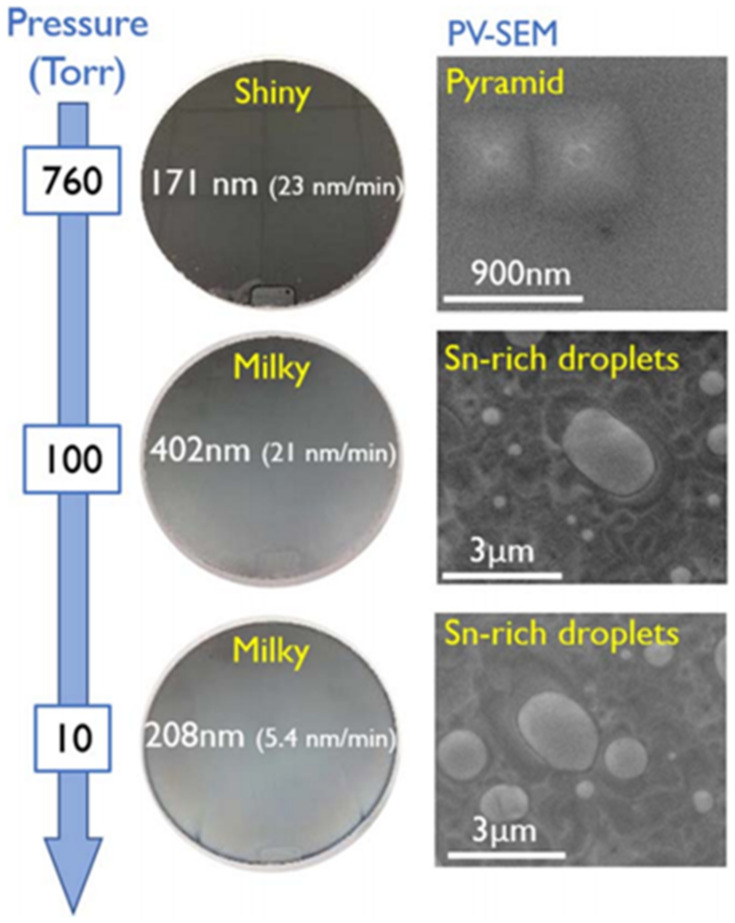
GeSn surface morphology vs. growth pressure (growth temperature: 320 °C; growth pressure: 10, 100, and 760 Torr; precursors: Ge_2_H_6_ and SnCl_4_). Reproduced with permission from [119], IOP Publishing, 2018.

For GeSn growth in a UHVCVD chamber [158,159,160,161], growth pressure is usually kept in the range of 1 × 10^−4^–2.5 × 10^−4^ Torr, and Sn content rises with decreasing growth temperatures. Even when different combinations of precursors (SnD_4_/Ge_2_H_6_, SnD_4_/Ge_3_H_8_, and SnD_4_/Ge_4_H_10_) are chosen, similar Sn content variation trends are observed (Figure 10). However, growth temperatures with different precursor combinations are varied; the lowest reported growth temperatures for SnD_4_/Ge_2_H_6_, SnD_4_/Ge_3_H_8_, and SnD_4_/Ge_4_H_10_ are 250, 350, and 150 °C, respectively [145,146,161]. Different from UHVCVD, pressures for GeSn growth in LPCVD and APCVD chambers have been found to range from 10 to 760 Torr [115,116,117,150]. The surface morphology of a layer GeSn grown by APCVD is shown in Figure 11, where surfaces are milky and pyramidical defects are observed at pressures of 10 and 100 Torr; this issue can solved by further increasing the growth rate (keep the SnCl_4_ constant and increase the Ge_2_H_6_ gas flow).

For GeSn APCVD growth at a temperature of 320 °C, pressure was found to be a main factor in the growth of high-Sn-content GeSn materials (the achieved Sn contents at 10 and 760 Torr were 16.7% and 6.6%, respectively) [119]. For LPCVD growth at 120 Torr and 320 °C, the Sn content for GeSn was almost the same as that of APCVD.

#### 2.3.2. Carrier Gas Effect on GeSn Growth

**Figure 12:**
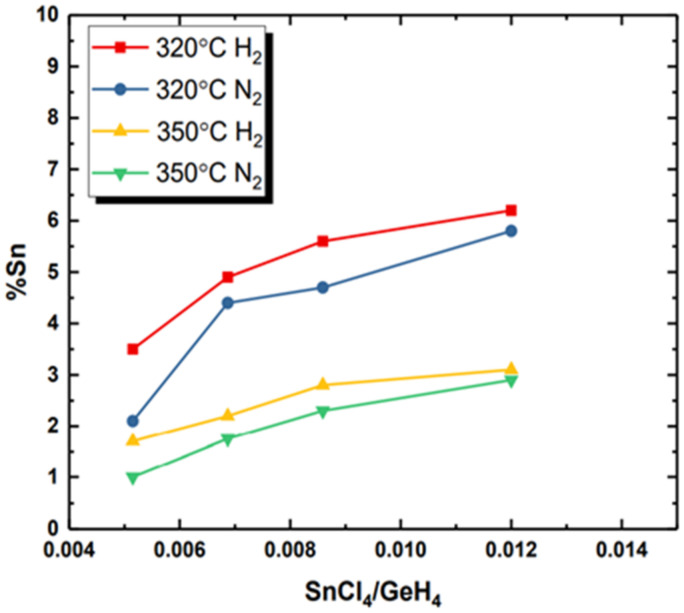
Sn content vs. SnCl_4_/GeH_4_ ratio (growth temperatures: 320 and 350 °C; carrier gases: N_2_ and H_2_; precursors: GeH_4_ and SnCl_4_). Reproduced from [153], open access by ASU library.

The effect of carrier gas on GeSn CVD growth is important and of great significance for the good mixing of precursor gases in a CVD chamber [105,152,153]. In contrast to pure Ge growth, the GeSn CVD growth mechanism has changed due to the introduction of Sn precursors, which have made GeSn CVD growth more complex. In several instances, a thickness reduction or an absence of GeSn occurs when choosing N_2_ as the carrier gas; this indicates that the growth rate has already changed and is below the etching rate from HCl. Furthermore, the Sn content of GeSn grown with an N_2_ carrier gas is different from that grown with an H_2_ carrier gas (Sn% difference is usually approximately 1%; see Figure 12). This Sn content reduction may be mainly attributed to the lower growth rate found when using N_2_ as the carrier gas.

#### 2.3.3. Strain Relaxation Effect on GeSn CVD Growth

**Figure 13:**
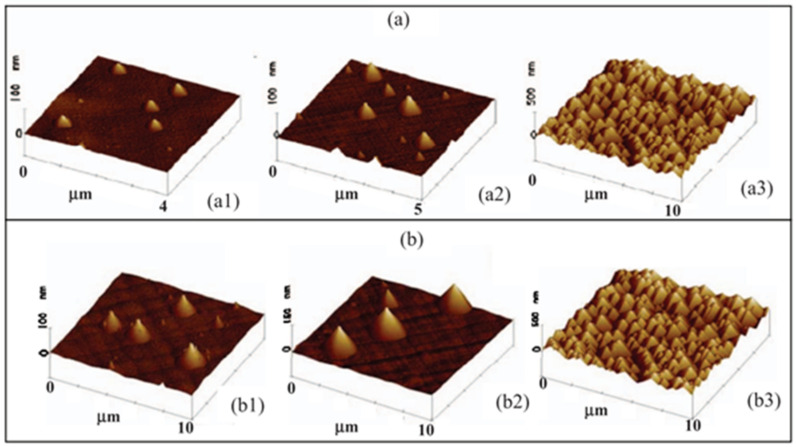
AFM images of (**a**) GeSn with an Sn content of 6.4%; the strain relaxations for (**a1**), (**a2**), and (**a3**) are 8%, 33%, and 75%, respectively. (**b**) GeSn with a strain relaxation of 75%; the Sn contents of (**b1**), (**b2**), and (**b3**) are 12.6%, 8.1%, and 6.4%, respectively. Reproduced with permission from [162], IOP Publishing, 2012.

F. Gencarelli et al. discovered a composition-dependent strain relaxation mechanism, and they found that high-Sn-content materials show a classical strain relaxation behavior [162]. Their AFM results showed that the island size and density of their low-Sn-content GeSn layers increased with strain relaxation degree (Figure 13) for the following reasons: higher amounts of Sn precursors were needed for high-Sn-content GeSn growth, extra Cl doses were exposed to the surface of GeSn and thus likely avoided Ge–Sn diffusion, Cl atoms could be regarded as the surfactants to mediate the enhancement of island size and density.

**Figure 14:**
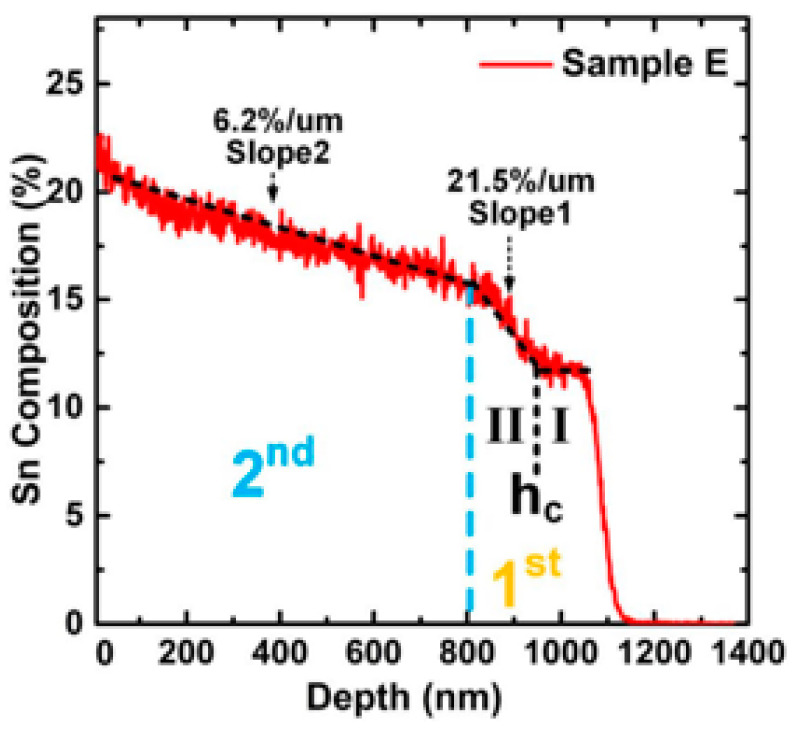
SIMS result for the GeSn sample with an Sn content of up to 22.3% (the maximum Sn contents for regions I, II, and III were 11.9%, 15.5%, and 22.3%, respectively). Reproduced from [113], Springer Nature, open access, 2018.

Later, high-quality GeSn with a world-record high Sn content of 22.3% was crafted after the discovery of strain-relaxation-enhanced (SRE) GeSn CVD growth mechanism [113], thus showing that compressive strain is the primary limiting factor for achieving greater Sn incorporation under an Sn oversaturation condition (Figure 14). In this research, the following growth strategy was proposed: (i) for first GeSn layer growth, they used a growth recipe of 9–12% Sn (the Sn content ranged from 8.8 to 11.9%); (ii) for second GeSn layer growth, they used the same growth recipe, and the SnCl_4_ flow fraction increased by ~8% compared to the first GeSn layer (the Sn content ranged from 12.5 to 16.5%); and (iii) for third GeSn layer growth, they used the same growth recipe, and the SnCl_4_ flow fraction increased by ~8% compared to the second GeSn layer. It should be noted that the grading rate of Sn incorporation was well-designed to suppress the growth breakdown. Inspired by the discovery of the SRE GeSn CVD growth mechanism, S. Assali et al. grew a high-quality GeSn layer with 15% Sn using low pressure chemical vapor deposition (LPCVD) in 2018 [115,116].

### 2.4. Doping for GeSn

Mainstream GeSn doping technologies, such as ion implantation and in situ CVD doping, have been intensively studied for future electronics and photonics applications. Low contact resistivity plays a vital role in the creation of high-performance devices. Table 1 presents a summary of reported B, BF_2_^+^, and P-doped GeSn via ion implantation in terms of year, institution, Sn content, doping type, doping concentration, activation temperature, and contact metal. Additionally, Table 2 and Table 3 present summaries of B-doped GeSn, P-doped GeSn, and As-doped GeSn in terms of year, institution, Sn content, doping type, doping concentration, contact metal, and contact resistivity.

#### 2.4.1. Ion Implantation for GeSn

Ion implantation is a widely used technique for doping semiconductor materials, and its advantages include low-temperature operation, precise dose control, good uniformity, and extremely small lateral diffusion. The research and development of GeSn’s ion implantation technology is also of great significance for future device application. So far, researchers have carried out extensive research into GeSn ion implantation technology (although most GeSn has been grown in MBE chambers, which are also significant).

Phosphorus has been widely adopted for ion implantation to achieve efficient N-type doping in GeSn layers because its doping concentrations usually ranges from 2.1 × 10^19^ to 2.1 × 10^21^ cm^−3^. For the P-type doping, there are two options: boron and BF_2_^+^. The highest P-type doping concentration can reach up to 1 × 10^20^ cm^−3^.

#### 2.4.2. In Situ GeSn CVD Doping

Optoelectronic devices, such as GeSn LEDs, GeSn lasers, and GeSn detectors, generally need highly doped GeSn for efficient carrier recombination and low contact resistance. Electronic devices, such as GeSn MOSFETs, GeSn TFETs, GeSn FinFETs, and GeSn GAAFETs (gate-all-around), require lower ohmic contacts, higher dopant concentrations, and selective doping. The use of in situ doping technology for GeSn is an attractive route for improving the performance of optoelectronic and electronic devices because it enables the doping of GeSn at low temperatures with a high doping efficiency and selective doping. Indeed, GeSn transitions from an indirect to direct bandgap material with an Sn content as high as 10%, and this property has led to research interest in Si-based, high-efficiency light sources. The first electrically injected GeSn lasers were recently demonstrated with Sn contents of 11% and 15%. It is definitely true that we require better solutions to create direct bandgap, high-quality doped GeSn, and the selection of an appropriate reaction doping gas and the optimization of epitaxial process are vital for this purpose. To this end, the growth of B-doped GeSn, P-doped GeSn, and As-doped GeSn using CVD has been reported by several institutions, as summarized in Table 2. However, there are several key points to consider: (I) Sn loss occurs for B-doped GeSn CVD growth, indicating that there is a competition between Sn and B atoms [150,185]; (II) excess partial pressure for PH_3_ contributes to poor material quality due to P segregation; (III) B_2_H_6_ partial pressure has no degradation effect on material quality, though it increases the activation doping concentration; (IV) more P could be incorporated into Ge and GeSn by using high order precursors; (V) boron δ-doping layers are helpful for highly doped GeSn growth, and the maximum B concentration can reach up to 1 × 10^20^ cm^−3^; and (VI) the doping efficiency of As-doped GeSn is better than that of P-doped GeSn [110].

**Table 2:** Summary of reported B-doped GeSn, P-doped GeSn, and As-doped GeSn in terms of year, institution, Sn content, doping type, doping concentration, and contact metal.

Year	Institution	Sn Content (%)	N-Type	P-Type	Doping Concentration (cm^−3^)	Contact Metal	Ref.
**2009**	ASU	2	√	——	P: 1 × 10^20^	Cr/Au	[57]
**2011**	IMEC	8	——	√	B: 1.7 × 10^19^	——	[150]
**2013**	KTH Royal Institute of Technology	9.4	√	√	B: 5 × 10^18^ P: 1 × 10^20^	——	[186,187]
**2016**	PGI 9	8 and 11	√	√	B: 2 × 10^19^ P: 1 × 10^20^	——	[188]
**2016**	PGI 9	8.5 and 15	√	√	B: 4 × 10^18^ P: 7.5 × 10^19^	——	[189]
**2017**	ASM	9	√	——	As: >2 × 10^20^	——	[110]
**2017**	ASM and IMEC	1.4	——	√	B: 2 × 10^20^	——	[190]
**2018**	National Taiwan University	10	√	√	B: Sn loss P: No Sn loss	——	[191]
**2019**	National Taiwan University	>12	——	√	B: >1 × 10^21^	Ti	[192]
**2019**	Leti	10 and 15	√	——	P: 5 × 10^20^	——	[193]
**2020**	National Taiwan University	2, 4.7, and 13	——	√	B: 2.1 × 10^20^ for 2% Sn	Ti	[194]
**2020**	Leti	6.5	√	√	B: 5.2 × 10^19^ P: 2.2 × 10^20^	——	[195]
**2020**	National Taiwan University	4.7	——	√	B: 1.9 × 10^20^	Ti	[196]
**2021**	National Taiwan University	9	√	——	P:1.3 × 10^20^	Ni	[197]

**Table 3:** Summary of reported B-doped GeSn, P-doped GeSn, and As-doped GeSn in terms of institution, Sn content, doping type, doping concentration, and contact metal.

Year	Institution	Sn Composition (%)	N-Type	P-Type	Doping Concentration (cm^−3^)	Contact Metal	Contact Resistivity (Ω·cm^2^)	Ref.
**2014**	Institute of Microelectronics, Chinese Academy of Sciences	4	——	——	——	Ni	——	[187]
**2018**–**2020**	National Taiwan University	9	√	——	P:1.3 × 10^20^	Ni	1.5 × 10^–7^	[191,192,194,196,197]
		2, 4.7, and 13	——	√	B: 2.1 × 10^20^ for 2% Sn	Ti	4.1 × 10^–10^ for 2% Sn	
		4.7	——	√	B: 1.9 × 10^20^	Ti	1.1 × 10^–9^	
		>12	——	√	B: >1 × 10^21^	Ti	4.1 × 10^–10^	
		10	√	——	P: 1.3 × 10^20^	Ni	1.1 × 10^–7^	
		9	√	√	B: 4 × 10^17^ P: ——	Ni	3.8 × 10^–8^	
**2020**	Leti	6.5	√	√	B: 5.2 × 10^19^ P: 2.2 × 10^20^	——	——	[195]
**2020**	Université de Montréal	11	√	√	B: × 10^19^ As: × 10^20^	——	——	[198]
**2019**	University College Cork	8	——	——	——	Ti, Ni, and Pt	——	[199]
**2013**–**2019**	NUS	5, 7, and 8	——	√	Ga: 3.4 × 10^20^	Ti	4.4 × 10^−10^ for 7% Sn	[165,166,167,168,169,170,171,172,173,200,201,202,203]
		8.5	——	√	Ga: 3.2 × 10^20^	——	——	
		5	——	√	Ga: ——	Ti	9.3 × 10^−10^	
		5	——	√	Ga: ——	Ni	2 × 10^−10^	
		5	——	√	Ga: 1.6 × 10^20^	Ni	1.4 × 10^−9^	
		2.4	√	——	P: 2.1 × 10^19^	Al	4 × 10^–3^	
**2012**	NUS and CAS-IOS	5.3	——	√	BF_2_^+^: 5.7 × 10^20^	Ni	1.6 × 10^–5^	[204]
**2015**– **2020**	CAS-IOS	7	√	——	Sb: 5 × 10^20^	Ni	1.3 × 10^–6^	[179,182,183,205,206]
		8	√	——	Sb: 3 × 10^19^	Ni/Al	6.2 × 10^–5^	
		7	√	——	Sb: 5 × 10^19^	Ni	1.3 × 10^–6^	
		3.2	√	——	P: 7.64 × 10^19^	Ni/Al	2.26 × 10^–4^	
		7	——	√	P: 2.44 × 10^19^	Ni/Al	1.9 × 10^–6^	

#### 2.4.3. GeSn Ohmic Contact

**Figure 15:**
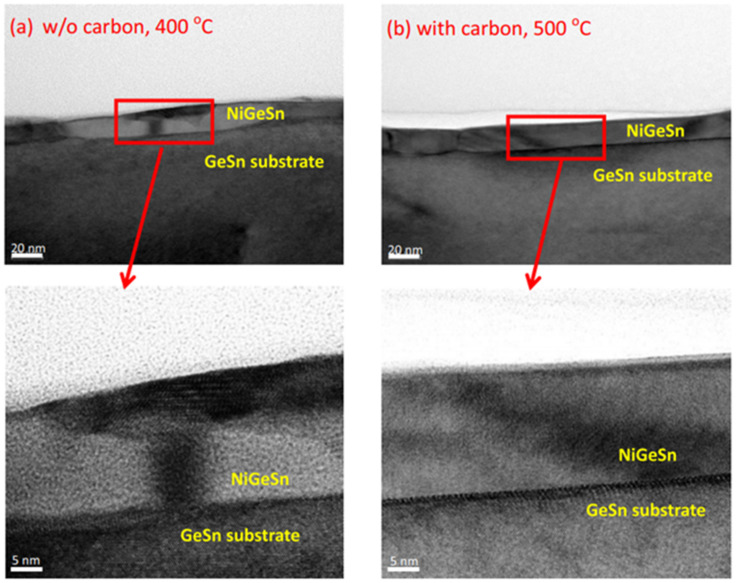
TEM images for Ni–GeSn interface (**a**) annealing at 400 °C without C and (**b**) annealing at 400 °C with C. Reproduced from [187], IOP Publishing, open access, 2015.

Among the summarized GeSn contact works is that of Henry. H. Radamson et al., who proposed a novel method to improve the thermal stability of the Ni–GeSn contact. It is well-known that carbon stabilize NiSiGe materials, so after GeSn growth, they implanted C into GeSn. In Figure 15, we can see that the NiGeSn film with C was more uniform than the NiGeSn film without C. Characterization results indicated that the presence of C not only led to the improved thermal stability but also tended to change the preferred orientation of NiGeSn [187]. A comparison work with different contact metals (10 nm of Ni, Ti, and Pt) [199] showed that Ni–GeSn was the most promising candidate due to its low sheet resistance and low formation temperature (below 400 °C). Moreover, Pt–GeSn showed better behavior in terms of thermal stability compared to Ni–GeSn and Ti–GeSn. Because Sn loss occurs during B-doped GeSn CVD growth, it is still challenging to create low contact resistivity p-type GeSn contacts with high Sn contents, a challenge that is particularly critical for GeSn lasers and GeSn TFETs [207,208].

## 3. Research Progress for GeSn Detectors

### 3.1. GeSn Photoconductive Detector

**Figure 16:**
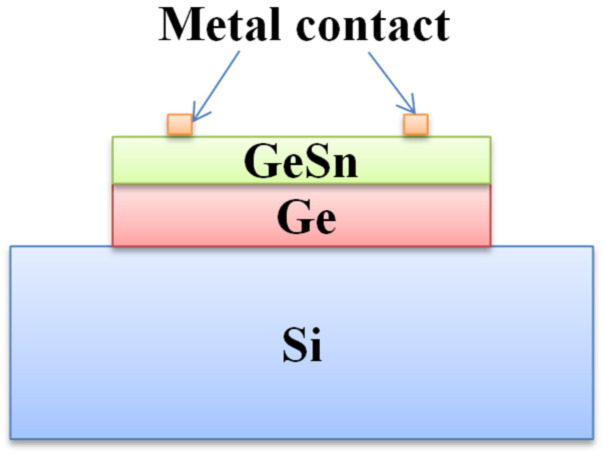
Cross-sectional schematic of a device structure for a GeSn photoconductive detector.

Photoconductive detector, which can also be defined as metal–semiconductor–metal (MSM) detector, is regarded as the simplest structure to achieve detection. In this type of structure, two Schottky junctions are designed and the total layer structure does not require any doping. Therefore, it can only work at a high bias voltage due to the existence of high contact resistance. However, the capacitance of a photoconductive detector is quite low, which is helpful for high-speed detection. Based on the photoconductive structure, researchers have put great effort into GeSn photoconductive detectors (Figure 16). Table 4 shows the reported performance levels of GeSn photoconductive detectors grown by CVD technology.

**Figure 17:**
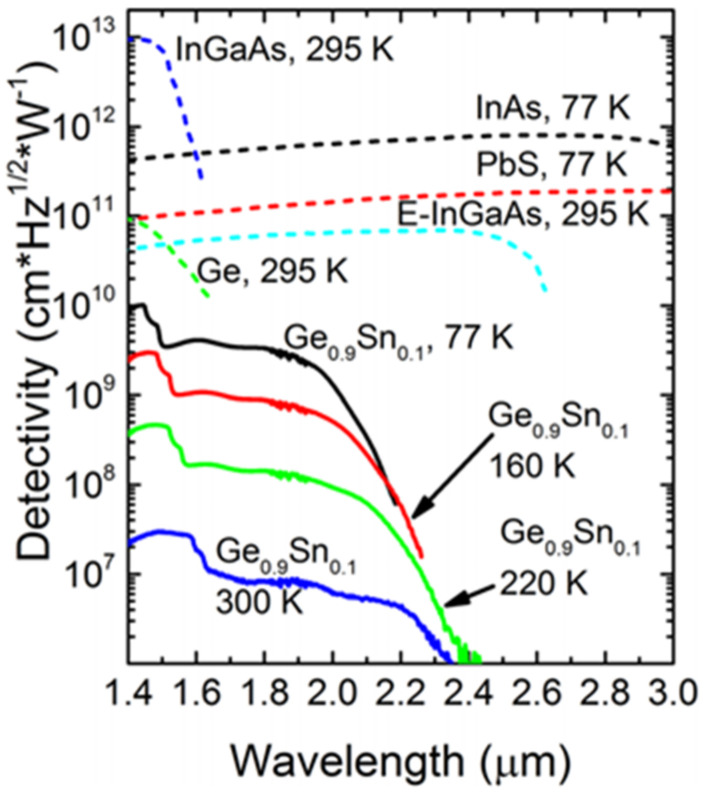
Specific detectivity for a Ge_0.9_Sn_0.1_ photoconductive detector at temperatures of 77, 160, 220, and 300 K. Reproduced from [60], OSA Publishing, open access, 2014.

As previously reported, IMEC mastered low-cost and commercially available cutting-edge GeSn growth technology in 2011 (Ge_2_H_6_/GeH_4_ precursor combination) [151]. Subsequently, they further grew a GeSn/Ge MQWs structure, and they also fabricated a photoconductive detector [58]. In 2014, Benjamin, R. Conley et al. reported the temperature-dependent spectral responses and detectivity of GeSn photoconductors with Sn contents ranging from 0.9 to 7% [59]. For a GeSn photoconductor with 7.0% Sn, a maximum wavelength response of 2100 nm was achieved. Experimental results showed that low-temperature responsivity was two orders of magnitude higher than room-temperature responsivity at 1550 nm, and the maximum specific detectivity was 1 × 10^9^ cm·Hz^1/2^/W at 77 K. In the same year, Benjamin, R. Conley et al. further extended the spectral response using a GeSn layer with 10% Sn [60]. The room-and low-temperature (77 K) wavelength cutoffs for the GeSn detector were found to be 2400 and 2200 nm, respectively. Maximum peak responsivity was observed as 1.63 A/W at 77 K due to photoconductive gain. More importantly, the specific detectivity was increased by about five times compared to the previously reported result (a GeSn photoconductor with 7.0% Sn), indicating that the material quality of the GeSn layer with 10% Sn was greatly improved (Figure 17).

**Figure 18:**
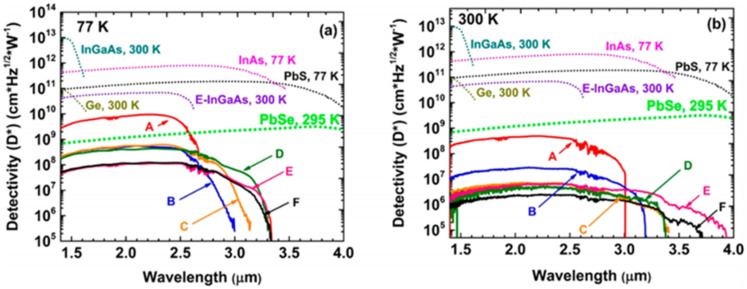
Specific detectivity for a GeSn photoconductive detector at the temperatures of (**a**) 77 K and (**b**) 300 K (the Sn contents for samples A–F were 12.5%, 15.9%, 15.7%, 17.9, 20.0%, and 22.3%, respectively). Reproduced with permission from [63], American Chemical Society, 2019.

In 2019, Huong Tran et al. reported a GeSn photoconductor with high Sn contents (the maximum Sn contents of the top GeSn layer were 12.5%, 15.9%, 15.7%, 17.9%, 20%, and 22.3%) [63]. As the Sn content increased, the cutoff wavelength shifted toward longer wavelength due to the bandgap shrinkage. From 77 to 300 K, the cutoff wavelengths were 3200–3650 nm for the GeSn photoconductor with 22.3% Sn. It is worth noting that this D* value was superior to that of a PbSe detector at the given wavelength range and was comparable to that of a commercial extended-InGaAs detector (4 × 10^10^ cm·Hz^1/2^·W^−1^) at the same wavelength range (Figure 18). Even at 300 K, the passivated device showed better results D* than the PbSe detector from 1500 to 2200 nm.

**Figure 19:**
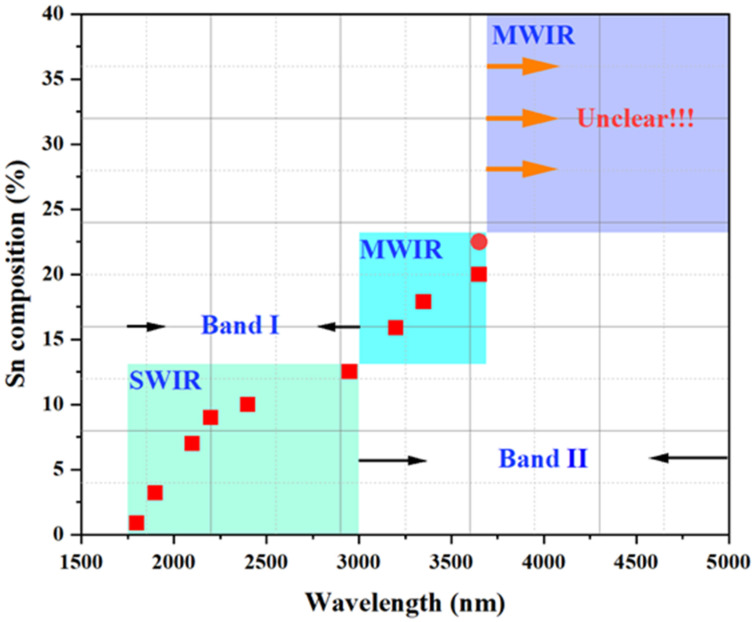
Sn content vs. wavelength of a GeSn photoconductive detector, indicating that GeSn is a promising absorber in SWIR and MWIR detection applications.

To enable a comprehensive overview of the use of GeSn photoconductive materials for infrared detection applications, Figure 19 illustrates the Sn content vs. cutoff wavelength for reported GeSn photoconductive detectors. For GeSn with an Sn incorporation of 0.9–12.5%, the photoconductive detector wavelength coverage was found to range from 1800 to 2950 nm, indicating that GeSn with Sn contents of up to 12.5% or 13% is very promising for SWIR applications. For GeSn with an Sn incorporation of 15.9–22.3%, the photoconductive detector wavelength coverage was found to range from 3200 to 3650 nm, suggesting potential mid wavelength infrared (MWIR) applications. For wavelengths from 3650 to 5000 nm, no detectors have been reported. However, GeSn photoconductive detector performance is limited by current growth technology and Sn distribution uniformity in total layer structures, which causes a low responsivity (the responsivity values are listed in the table above).

### 3.2. GeSn PIN Detector

**Figure 20:**
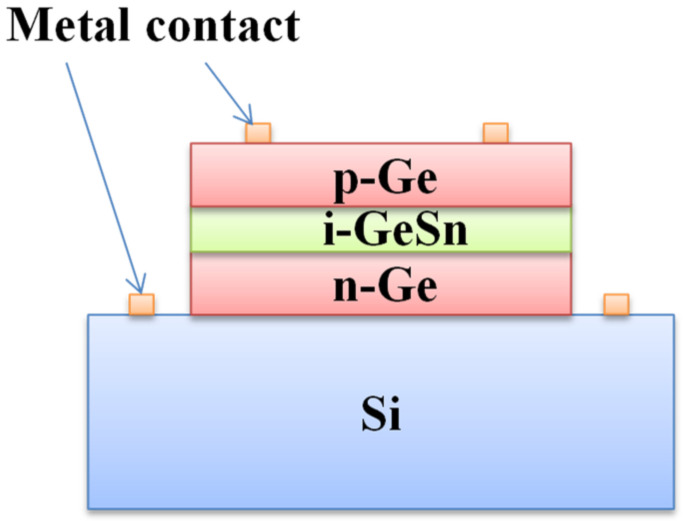
Cross-sectional schematic of typical device structure for a GeSn detector.

The PIN detector is the most common and widely used detector type for Si-based optoelectronics applications. One side of a PIN detector device is for p-type doping, and the other side is for n-type doping; as such, the built-in electric field is able to locate the intrinsic region [18]. A typical cross-sectional schematic diagram of a GeSn PIN detector is shown in Figure 20, and the major device performance values for reported GeSn PIN detectors are summarized in Table 5.

**Figure 21:**
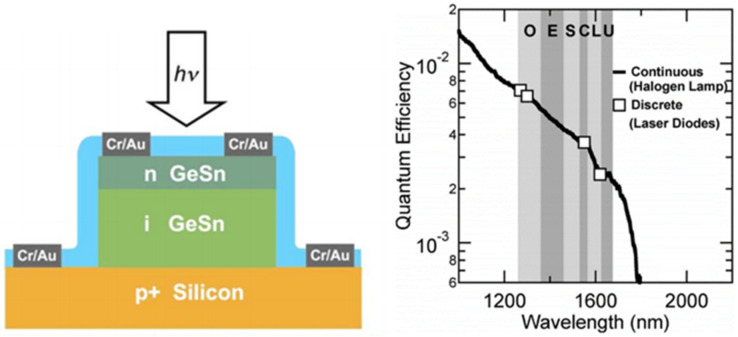
Cross-sectional schematic of a GeSn photodetector and its quantum efficiency as a function of wavelength. Reproduced with permission from [57], AIP Publishing, 2009.

In 2009, Jay Mathews et al. demonstrated the first GeSn photodetector with 2% Sn content; 350 nm Ge_0.98_Sn_0.02_ was directly grown on a B-doped Si (100) substrate in an UHVCVD system (the carrier concentration in the Si wafer was 4.3 × 10^19^ cm^−3^) [57]. Three cycles of post-growth annealing were carried out to decrease the TDDs in Ge_0.98_Sn_0.02_. Afterwards, n-doped Ge_0.98_Sn_0.02_ was further deposited, and its carrier concentration was found to be approximately 7.5 × 10^19^ cm^−3^. Using the abovementioned layer structure, a circular GeSn photodetector was fabricated. To evaluate the quantum efficiency of the Ge_0.98_Sn_0.02_ photodetector, the circular mesa was continuously illuminated via a halogen source and 1270, 1300, 1550, and 1620 nm lasers. The Ge_0.98_Sn_0.02_ detector quantum efficiencies were higher than those in comparable pure Ge device designs processed at low temperatures (Figure 21). Additionally, the wavelength cutoff was extended to at least 1750 nm, which means that a GeSn photodetector with 2% Sn content can cover the entire telecommunication band.

**Figure 22:**
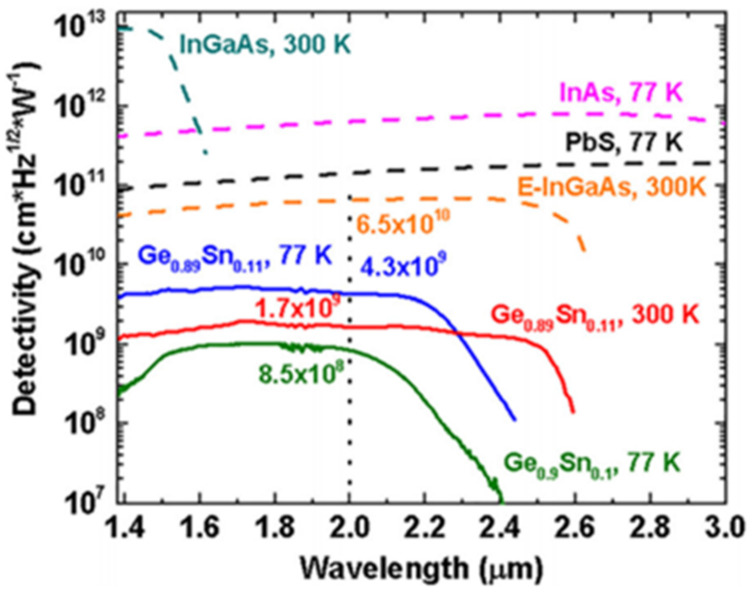
Specific detectivity for a Ge_0.89_Sn_0.11_ photodetector at the temperatures of 77 and 300 K. Reproduced with permission from [65], AIP Publishing, 2018.

In 2018, Huong Tran et al. fabricated GeSn photodetectors with 700 nm thick GeSn layers using the p–Ge/p–Ge_0.91_Sn_0.09_/i–Ge_0.89_Sn_0.11_/n–Ge_0.89_Sn_0.11_/n–Ge layer structure (all layers were grown by RPCVD) [65]. In order to obtain detailed and accurate external reading of quantum efficiency, all GeSn photodetectors were illuminated with a 2000 nm laser. Room-temperature peak responsivity and external quantum efficiency were measured to be 0.32 A/W at 2000 nm and 20%, respectively. When the GeSn detector was illuminated by a 1550 nm laser, its external quantum efficiency reached up to 22%. Different from the previously reported thin film photoconductor, the thick film photoconductor showed an extended wavelength cutoff (2650 nm) due to the reduced strain relaxation and enhanced light absorption in the thick GeSn film. Nevertheless, the peak specific detectivity for the GeSn detector was compared to other commercial infrared detectors at a wavelength range from 1400 to 3000 nm, which showed that peak specific detectivity of the GeSn detector at 2000 nm was only one order of magnitude lower than that of the extended-InGaAs detector (Figure 22). To improve device performance, Xu S, et al. attempted to create a GeSn/Ge MQW detector [67,68], a GeSnOI detector [69], and a photon-trapping microstructure GeSn/Ge MQW detector [209].

**Figure 23:**
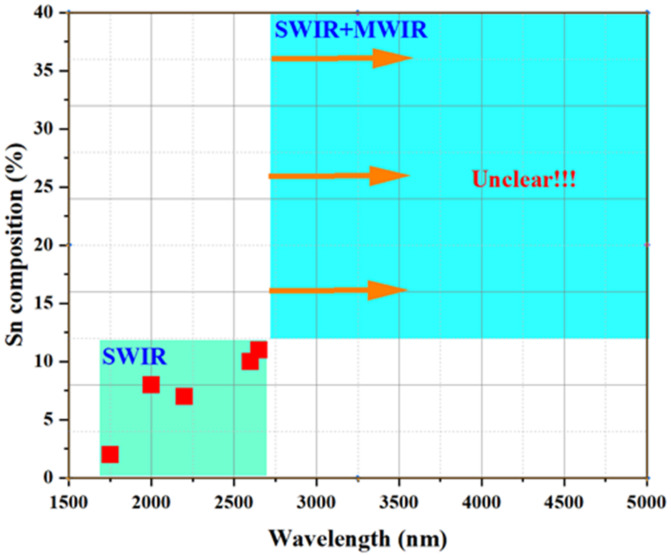
Sn content vs. cut-off wavelength of the GeSn PIN detector.

Figure 23 summarize the Sn content vs. cut-off wavelength for a reported GeSn PIN detector. For GeSn with an Sn incorporation of 2–11%, the PIN detector wavelength coverage was found to range from 1750 to 2650 nm, indicating that a GeSn PIN detector is very promising for SWIR applications. Due to the limitations of growth technology, PIN detectors at wavelengths from 2650 to 5000 nm have yet to be reported.

## 4. Research Progress for GeSn Lasers

Since Si-based high-efficiency light sources comprise the technical bottleneck for Si-based monolithic optoelectronic integration, researchers have conducted extensive research into Ge and GeSn lasers. Ten years ago, the rapid development of the GeSn CVD growth technique enabled researchers from MIT to demonstrate optically injected and electrically injected Ge lasers at room temperature. The lasing thresholds of these laser devices were very high, which made it difficult to achieve efficient lasing. As a result, more attentions has been paid to the GeSn material due to its direct bandgap property. In this section, we review the latest research on GeSn lasers with different optical cavities, as well as their device performance.

### 4.1. Optically Injected GeSn Lasers

#### 4.1.1. Optically Injected GeSn Laser with FP Cavity

**Figure 24:**
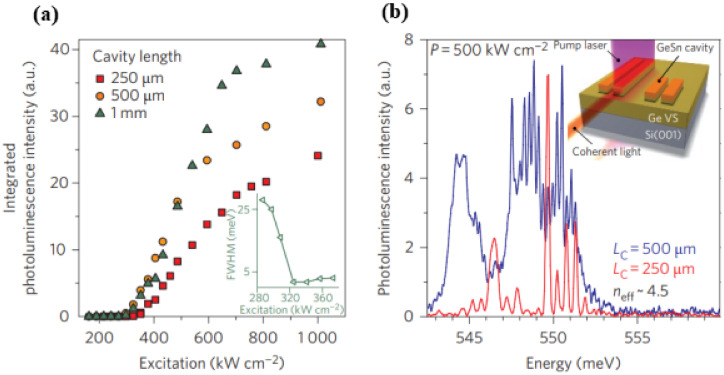
(**a**) Integrated PL intensity vs. excitation power density for a GeSn FP cavity laser with different cavity lengths; (**b**) high-resolution laser spectra for a GeSn laser with cavity lengths of 250 and 500 μm. Reproduced with permission from [70], Springer Nature, 2015.

Based on the GeSn optical gain medium, the world’s first optically injected FP cavity GeSn laser was demonstrated at a low temperature [70]. The typical threshold power densities of FP cavity GeSn lasers with cavity lengths of 1 mm, 500 μm, and 250 μm were maintained between 300 and 330 kW/cm^2^ (Figure 24a). When the optically injected power density was above its threshold power density, the full width half maximum of the optical emission spectrum was dramatically reduced and the intensity was significantly increased; when the optical injection power density increased to 650 kW/cm^2^, the threshold curve tended to be flat (possibly due to a self-heating effect) (Figure 24a). When the optically injected power density increased to 1000 kW/cm^2^, the maximum lasing temperature for the GeSn laser with 12% Sn content was 90 K. Figure 24b shows high-resolution laser spectra that indicate the performance of a GeSn laser under multi-mode operation.

**Figure 25:**
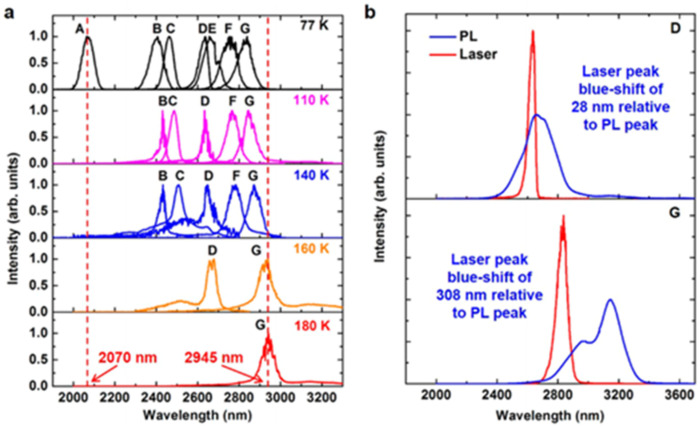
(**a**) GeSn laser spectra for samples A–G; (**b**) comparison of the PL and laser spectra of samples D and G. Reproduced with permission from [73], American Chemical Society, 2017.

In 2017, Joe Margetis et al. systematically studied the performance of optically injected GeSn lasers with different Sn contents [73]; the Sn contents of samples A–G were 7.3%, 9.9%, 11.4%, 14.4%, 15.9%, 16.6%, and 17.5%, respectively, and the maximum operation temperatures of samples A–G were 77, 110, 140, 160, 77, 140, and 180 K, respectively (Figure 25). Except for sample A (lower Sn content) and sample E (poor material quality), the samples could be lased at 140 K. It is worth noting that the maximum operation temperature of samples D and G were 160 and 180 K, respectively. The results showed that the operating temperature of the optically injected GeSn laser was closely related to the Sn content of GeSn, and the GeSn lasers with higher Sn contents possessed higher operating temperature (except for sample F because of its poor material quality). Therefore, increasing the Sn content in GeSn can effectively increase the operating temperature of the laser device. From the theoretical point of view, the main factors that affect the performance of laser devices are material gain, active layer thickness, device surface roughness, and non-radiative recombination. Therefore, there are differences in the operating temperatures of GeSn laser devices with different Sn contents.

**Figure 26:**
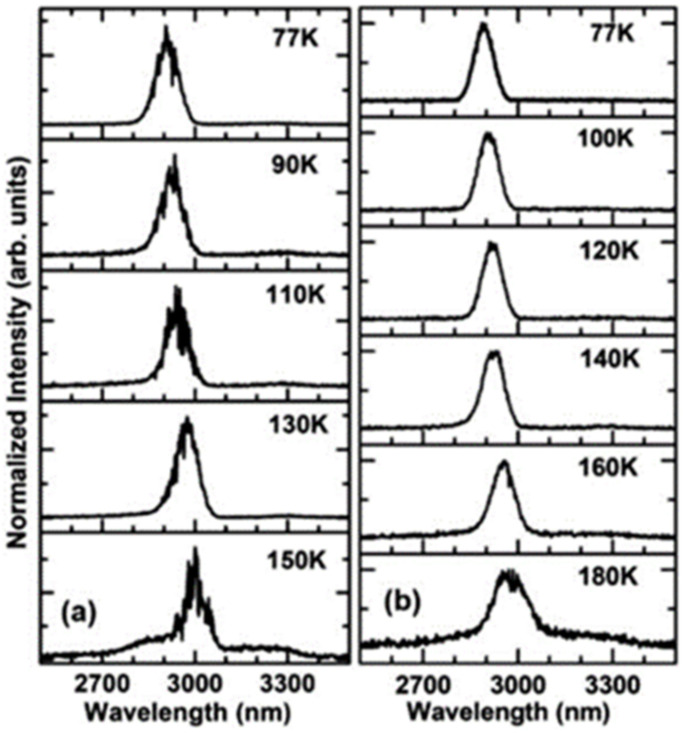
Temperature-dependent lasing spectra for a bulk GeSn laser with an Sn content of up to 22.3%; the optical injection sources were (**a**) a 1064 nm pulsed laser and (**b**) a 1950 nm pulsed laser. Reproduced from [75], OSA Publishing, open access, 2018.

Thanks to the discovery of the GeSn strain-relaxation-enhanced growth mechanism [88], researchers were able to increase the Sn content of GeSn to 22.3%. In this layer structure, the GeSn buffer layer is grown with a nominal recipe for 11% GeSn. When the thickness of the 11% GeSn layer reaches its critical thickness, internal strain in the GeSn layer gradually relaxes and more Sn atoms can be incorporated into the Ge lattice. Experimental results showed that the strain relaxation growth mechanism could lead to high-Sn-content GeSn alloys (higher than 22.3%). Later, Wei Dou et al. reported an optically injected bulk GeSn laser with an Sn content of up to 22.3% [75]; both 1064 and 1950 nm pulsed lasers were used for optical injection, and the maximum operating temperatures were 150 and 180 K, respectively (Figure 26).

**Figure 27:**
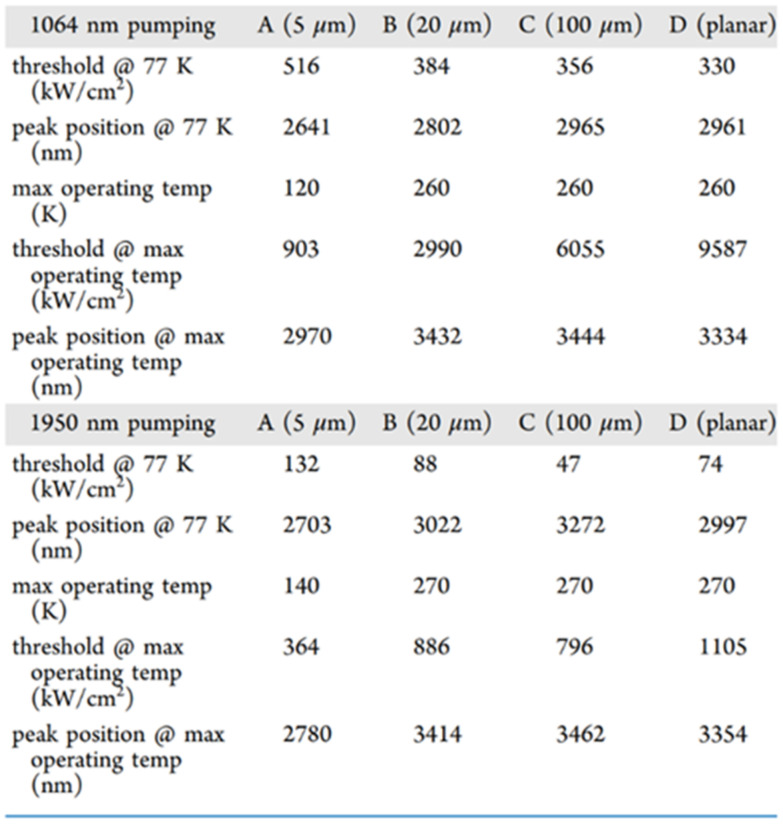
Summary of laser performance under 1064 and 1950 nm pulsed laser injection (the cavity widths for samples A, B, C, and D were 5 μm, 20 μm, 100 μm, and planar, respectively). Reproduced with permission from [76], ACS Publishing, 2019.

In 2019, Yiyin Zhou et al. researched optically injected GeSn lasers (an Sn content of 20%) with different waveguide widths [76]; 1064 and 1950 nm lasers were used for pulsed optical injection characterization (Figure 27). They concluded that the operation temperature for sample A was lower than those of the other samples (the laser operation temperatures under 1064 and 1950 nm pulsed injection were 120 and 140 K, respectively). Moreover, the threshold for sample A was relatively larger than those of the other samples (at 77 K, the thresholds under 1064 and 1950 nm optical pulsed injection were 516 and 132 kW/cm^2^, respectively). When the sample width was wider than 20 μm, the operation temperatures of the laser devices could be increased to 260 and 270 K under 1064 and 1950 nm optical pulsed injection, respectively. The reasons for this are as follows: (i) compared with the side wall surface recombination, free carrier absorption loss and non-radiative recombination were the dominant losses at higher temperatures; (ii) the stripe-shaped optical injection light beam had a Gaussian distribution, which may have resulted in absorption occurring in the middle of a wider waveguide (less absorption at the edge of the waveguide); and (iii) the optical confinement factor for sample D was lower, which led to a higher threshold.

**Figure 28:**
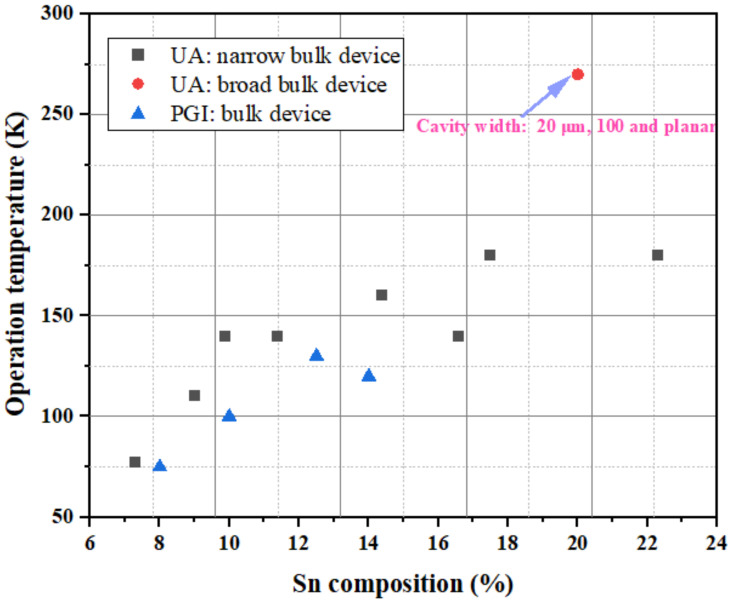
Maximum operation temperature vs. Sn content for optical pumped FP cavity GeSn laser (under pulsed 1064 nm laser) [70,74,75,76].

The simplest optical cavity is that of Fabry–Pérot, which consists of two parallel reflecting surfaces that allow coherent light to travel through the whole cavity. Due to the directness difference between GeSn alloys with different contents, we summarize the reported operation temperatures for GeSn with different Sn contents in Figure 28. Operation temperatures were found to increase with more Sn incorporation, indicating that operation temperature is closely related to the directness of GeSn. Different from narrow bulk devices, broad bulk devices (with a cavity width greater than 20 μm) possess higher operation temperatures, possibly due to the following two reasons: (1) they have higher optical gains, and (2) they are wider and thus have higher optical injection efficiencies. However, the operation temperature for a GeSn laser with 22.3% Sn incorporation was found to be the same as that of a GeSn laser with 17.5% Sn incorporation, which means that there were many point defects in the high-Sn-content GeSn layer. For clarification, we also summarize the devices performance for the published FP cavity optically pumped GeSn laser (Table 6). 

#### 4.1.2. Optically Injected GeSn Laser with WGM Cavity

**Figure 29:**
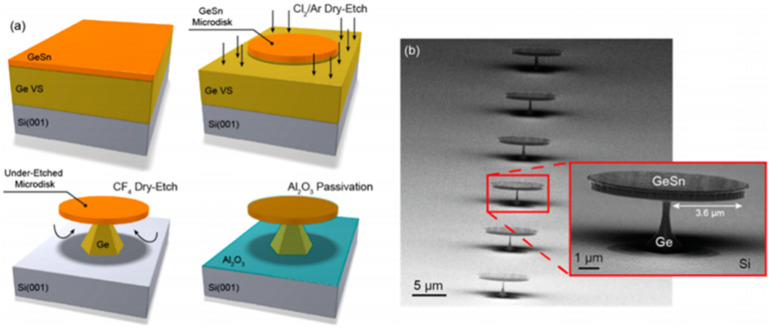
(**a**) Process flow for GeSn microdisk; (**b**) SEM image of GeSn microdisk with an Sn content of 12.5% (diameter was 8 μm). Reproduced with permission from [74], American Chemical Society, 2016.

**Figure 30:**
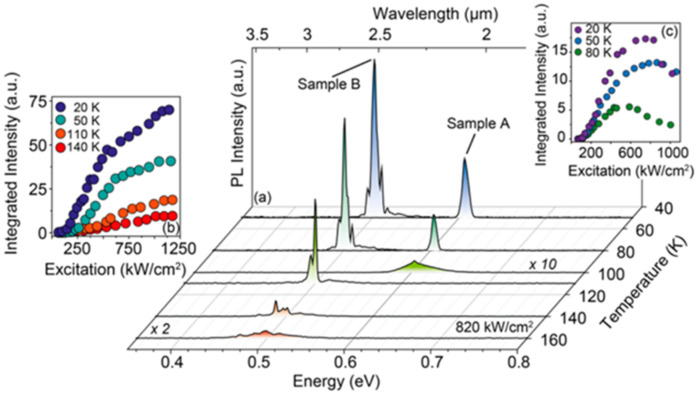
(**a**) Temperature-dependent lasing spectra for samples A and B (the Sn contents for samples A and B were 8.5% and 12.5%, respectively); (**b**,**c**) L–L curves for samples A and B, respectively. Reproduced with permission from [74], American Chemical Society, 2016.

In 2016, Daniela Stange et al. realized a self-suspending microdisk GeSn laser for the first time [74] (Figure 29). The laser spectrum is shown in Figure 30. It can be seen in the figure that the maximum working temperatures of samples A and B were 80 and 140 K, respectively. Compared with sample B, the lasing spectrum of sample A was blue-shifted due to its higher content. Although the operation temperature for sample A was lower than that of sample B, the threshold for sample A was lower than that of sample B (the thresholds of samples A and B were 125 and 220 kW/cm^2^ at 50 K, respectively).

**Figure 31:**
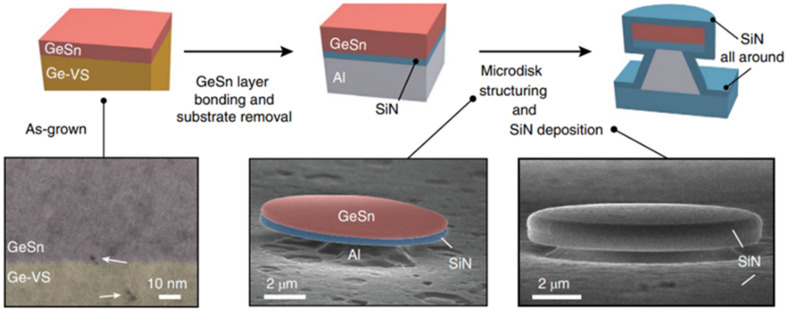
Fabrication process for a GeSn microdisk laser with SiN_x_ all-around. Reproduced with permission from [80], Springer Nature, 2020.

In 2020, Anas Elbaz et al. reported a CW optically injected GeSn microdisk laser with a low Sn content for the first time [80]. Compared with high-Sn-content GeSn, low-Sn-content GeSn has fewer internal point defects and better material quality. After its growth, a low-Sn-content GeSn layer was transferred to an Si substrate with SiN and Al layers. Then, the Si substrate, Ge buffer layer, and defective GeSn layers are removed; only 40 nm, high-quality, low-Sn-content GeSn was left. Finally, the transferred GeSn layer was patterned into independent GeSn/SiN microdisks supported by Al microdisk pillars (Figure 31). The lasing spectrum in Figure 31 shows the continuous wave light injection laser spectrum of a GeSn microdisk with a diameter of 7 μm at 25 K: below the threshold, a light emission spectrum with a wide half-width (red line) was obtained under an optical injection power of 0.5 mW; above the threshold, lasing emission characteristics were obvious under the optical injection power of 6.4 mW. Under the pulsed optical injection and CW light injection, the maximum operating temperatures of the laser device were 90 and 50 K, respectively.

**Figure 32:**
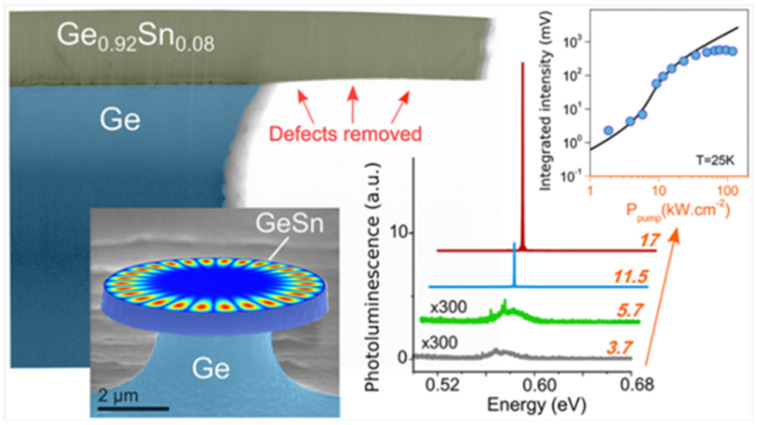
GeSn microdisk laser with removed defects under the disk. Reproduced with permission from [82], American Chemical Society, 2020.

In 2020, Anas Elbaz et al. created an optically injected GeSn microdisk laser after proper defect management [81,82], indicating that the threshold was greatly reduced compared to that of a GeSn microdisk laser without defect management (the lasing threshold reduction was 1 order of magnitude higher compared to examples in the literature). They also found that the maximum lasing temperature for the optically injected GeSn microdisk laser, with Sn contents ranging from 7% to 10.5%, only weakly depended on Sn content. Apart from the directness of the GeSn active region, the experimental results indicated that nonradiative recombinations and point defects are the main obstacles for high-temperature lasing (Figure 32).

**Figure 33:**
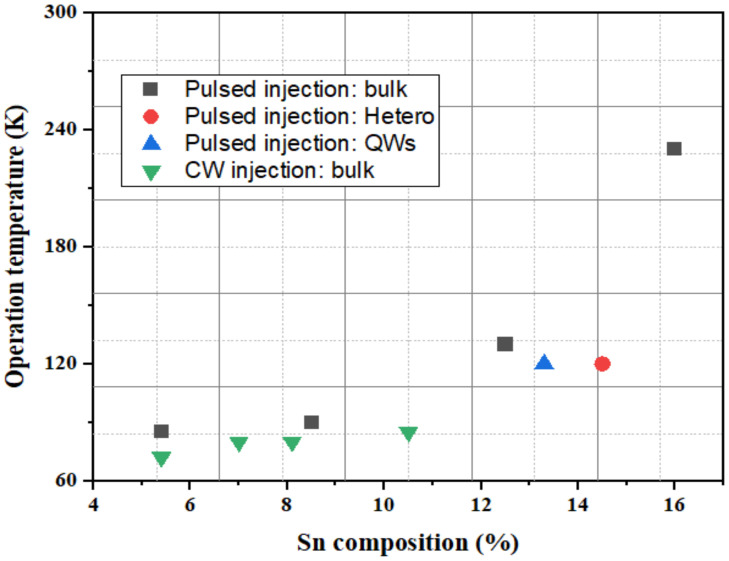
Maximum operation temperature vs. Sn content for an optically pumped WGM cavity GeSn laser (under pulsed 1064 nm laser).

The abovementioned GeSn microdisk laser results show that both pulsed and CW injection have been achieved (Table 7). Especially for CW lasing, this is the most direct evidence to verify that GeSn can withstand a CW injection test. To gain a better understanding of GeSn microdisk lasers, we summarize the operation temperatures for GeSn lasers with different Sn contents in Figure 33. For the pulsed injection, the operation temperature for the GeSn microdisk laser followed a similar trend to that of an FP cavity GeSn laser (the operation temperature increased with Sn content). However, the operation temperature for the heterostructure and quantum well GeSn laser was lower than that of bulk laser, suggesting that there is still room to improve the operation temperatures of heterostructure and quantum well lasers. For CW injection, it seems that operation temperature enhancement is not that sensitive to Sn content, though it brings efficient heat dissipation.

#### 4.1.3. Optically Injected GeSn Laser with Other Microcavities

**Figure 34:**
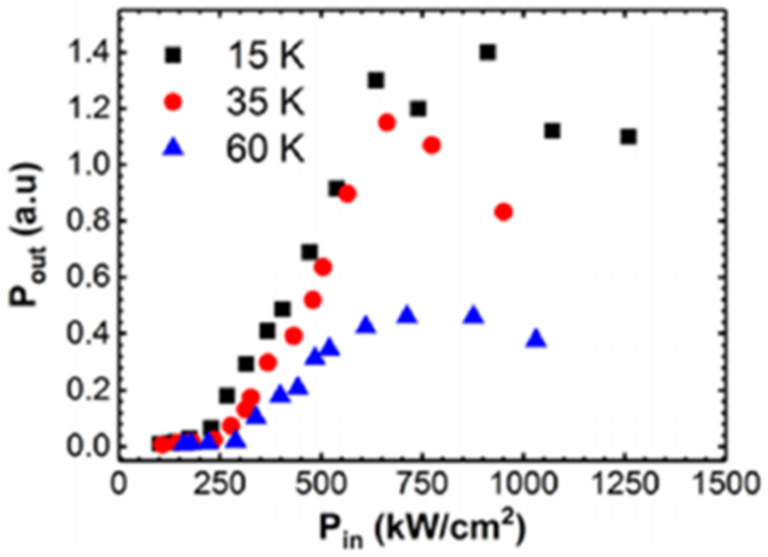
L–L curves for a photonic crystal GeSn laser with an Sn content of up to 16%. Reproduced with permission from [72], AIP Publishing, 2018.

In addition to those on FP cavity and microdisk cavity GeSn lasers, there have been publications on hexagonal photonic crystal (PC) and micro-bridge GeSn lasers. In 2018, Q.M. Thai et al. reported optically injected GeSn laser with 16% Sn content for the first time [72]. By introducing defects in the photonic crystal defect cavity (such as removing the central hole), the periodic structure around the photonic band gap were able to provide optical feedback to the microcavity. The experimental results showed that the maximum working temperature of the hexagonal photonic crystal GeSn laser was 60 K, and the threshold values at 15 and 60 K were 227 and 340 kW/cm^2^, respectively (Figure 34).

**Figure 35:**
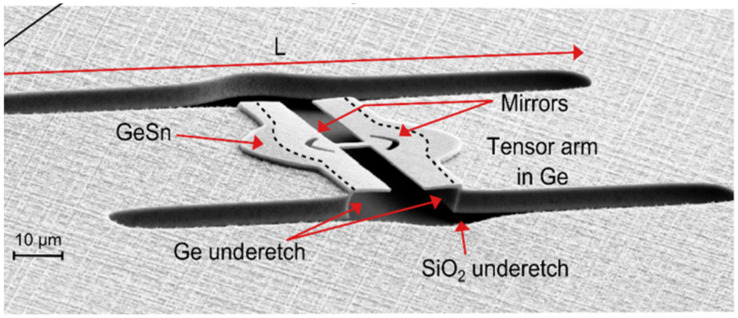
L–L curves for a micro-bridge GeSn laser with an Sn content of up to 16%. Reproduced with permission from [77], American Chemical Society, 2019.

In 2019, Jerémie Chrétien et al. explored a novel approach to create a direct bandgap GeSn material via strain redistribution, thereby controlling band structure and lasing wavelength [77]. Tensile-strained GeSn micro-bridge heterostructures were optically injected using pulsed 1064 and 2650 nm lasers (Figure 35), and the maximum operation temperature for the L = 75 μm micro-bridge structure laser was 273 K, which indicates that the operation temperature was very close to room temperature.

### 4.2. Electrically Injected GeSn Lasers

Different from optically injected GeSn lasers, electrically injected GeSn lasers are more suitable for practical applications. However, electrically injected GeSn lasers are more challenging to create due to the GeSn active gain medium having to overcome the extra metal absorption loss and more free carrier absorption (FCA) losses. Theoretical predication for the realm of possibility of electrically injected GeSn/SiGeSn lasers can be traced back to ten years ago, when Greg Sun et al. presented modelling and simulation results for an electrically injected SiGeSn/GeSn/SiGeSn double heterostructure laser with an Sn contents ranging from 6 to 12% [210]; they found that this type of laser requires cooling in the temperature range of 100–200 K after taking radiative, nonradiative, and Auger recombinations into consideration. Afterwards, Greg Sun et al. theoretically proposed that the lattice matched that of an Si_0.1_Ge_0.75_Sn_0.15_/Ge_0.9_Sn_0.1_/Si_0.1_Ge_0.75_Sn_0.15_ MQW laser [211], and they found that modal gain was very sensitive to the QW number in the active region and SiGeSn/GeSn/SiGeSn MQW could operate up to room temperature with a 2300 nm emission wavelength. For the SiGeSn/GeSn/SiGeSn MQW laser with 20 QWs, the optical confinement factor was calculated to be 0.74, and the modal gain was able to exceed 100/cm at a pumping current density of 3 kA/cm^2^, which was sufficient to attain room-temperature lasing.

**Figure 36:**
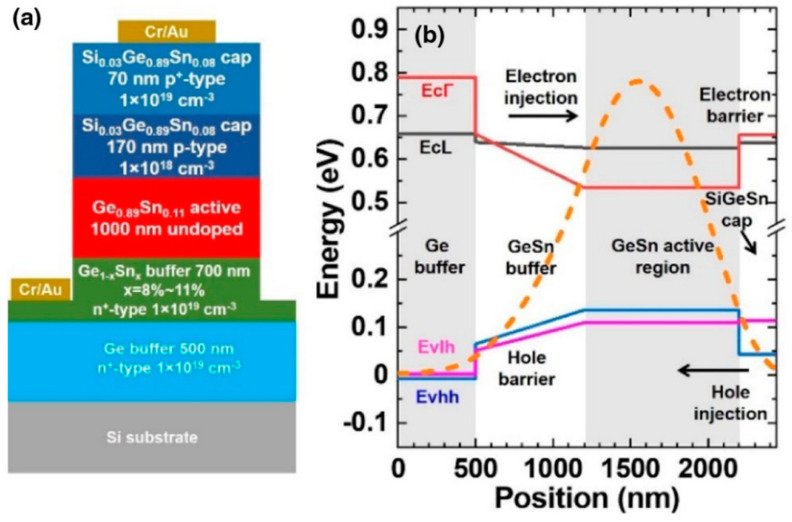
(**a**) Cross-sectional device structure for the first electrically injected FP cavity GeSn/SiGeSn laser; (**b**) calculated band structure and fundamental TE mode profile. Reproduced from [89], OSA Publishing, open access, 2020.

In 2020, Yiyin Zhou et al. reported the first electrically injected FP cavity GeSn/SiGeSn laser on Si with a lasing temperature of up to 100 K; its minimum threshold was approximately 598 A/cm^2^ [89,90] (Figure 36). This work was regarded as an essential achievement for Si-based on-chip light source in the development of Si-based OEICs. Later, the effects of cap layer, cap layer thickness, and Sn content in the active region on the operating temperature, threshold, and emission wavelength were further systematically studied [89,90]. Experimental results showed that: (I) an SiGeSn cap had a better optical confinement effect than a GeSn cap; (II) the optical confinement factor was improved via changing the SiGeSn cap layer thickness; and (III) the use of a GeSn laser with an Sn content of up to 15% did not significantly improve device performance.

## 5. GeSn Transistors

In addition to the rapid advancement of GeSn detectors and GeSn lasers grown by CVD technology, there have been some achievements in the field of GeSn transistors due to their mobility properties. In the hyper-scaling era, the quest for high-performance and low-power transistors is continuing and intensifying. One of the key technology enablers of these goals is that of channel materials with high carrier mobility and direct band gap structures [212,213]. GeSn films have emerged as the most promising candidate for next generation nano-electronic devices of computing due to their excellent properties, including ultrahigh hole mobility, band structures with direct and low band gaps, Si-based CMOS compatibility, and low thermal budget, all of which are of great importance for ultrahigh density devices and 3D integration in the hyper-scaling era. Anisotropy at the top of the GeSn valence band makes the effective mass of light hole rapidly decrease with increases of Sn content and the transport capacity rapidly increase. GeSn is a very promising channel material for the next generation pMOSFET, and its hole mobility is even higher than that of Ge. The hole mobility of Ge pMOSFET is increased by more than 10 times with respect to Si devices. In addition, compressive strain can improve the mobility of a GeSn channel by decreasing the effective mass of the hole carrier. GeSn is generally grown on Si substrates using Ge as the buffer layer, and GeSn subjects the Ge buffer layer to compressive strain since the Sn lattice constant is greater than that of Ge. As GeSn materials are compatible with Si-CMOS technology, a few research groups have studied GeSn-based transistors (Table 8 lists the reported transistors with CVD-grown GeSn layers).

**Figure 37:**
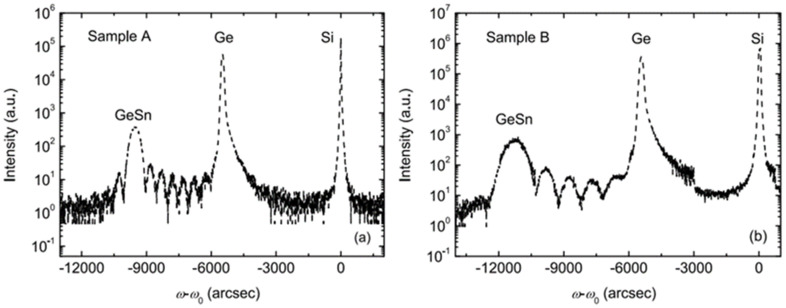
HR–XRD curves for GeSn samples with (**a**) 12% and (**b**) 15% Sn incorporation. Reproduced with permission from [217], IEEE, 2017.

**Figure 38:**
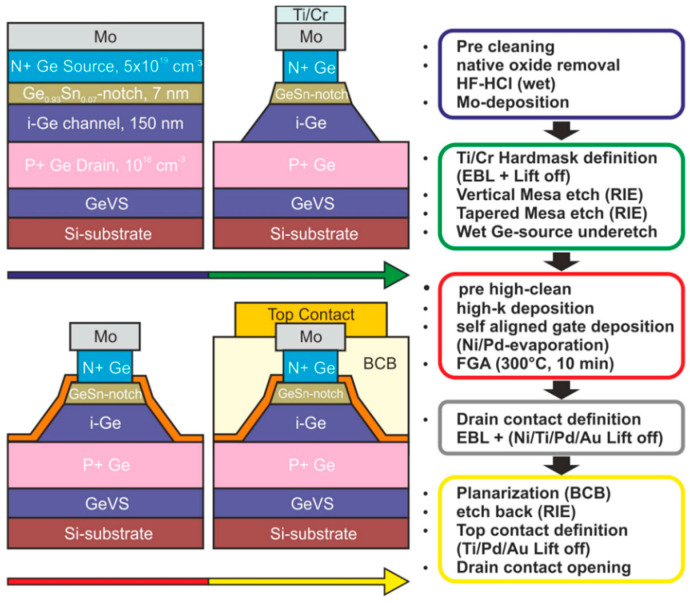
Process flow for Ge/GeSn vertical heterojunction pTFETs. Reproduced with permission from [99], IEEE, 2017.

**Figure 39:**
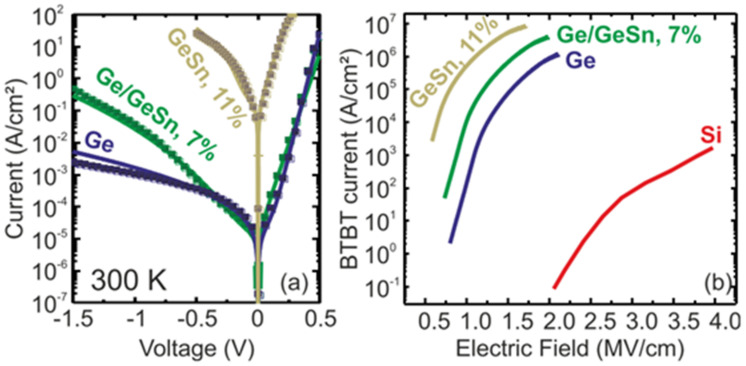
(**a**) RT current–voltage characteristics for GeSn p-i-n diode; (**b**) extracted BTBT current vs. electric field. Reproduced with permission from [99], IEEE, 2017.

Tunnel-field-effect transistors (TFETs) features subthreshold swings (SS) below 60 mV/decade at room temperature, which also enable a decreased power supply without discounting the off-current. Although Si-TFETs have been reported with SS below 60 mV/decade at low current, band-to-band tunneling (BTBT) is limited by its indirect bandgap property and low SS at high current. Therefore, researchers have investigated GeSn with high Sn contents (12% and 15% Sn incorporation; Figure 37) to create high-performance GeSn TFETs [217]. A higher Sn content enhances device performance, but the subthreshold swing is affected by the increased leakage level. For ultrasmall supply voltages, the device structure should be optimized to improve device characteristics. Using Ge/GeSn heterostructure pTFETs led to the improvements of the BTBT rate. Thus, higher on-current and lower off-current were achieved simultaneously. Christian et al. reported the fabrication and characterization of Ge/GeSn pTFETs (Figure 38), and they recorded a low accumulation capacitance of 3 μF/cm^2^ [99]. Moreover, their room-temperature (RT) current–voltage characteristics showed that the Ge/GeSn pTFETs with the 11% Sn content had the highest BTBT current (Figure 39).

**Figure 40:**
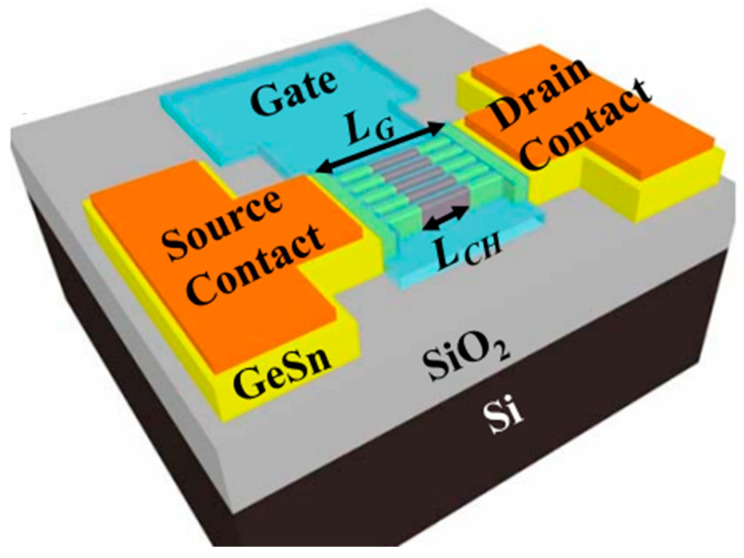
3D diagram and highlights of the first GeSn FinFETs grown on a GeSnOI substrate. Reproduced with permission from [93], IEEE, 2018.

To suppress the short channel effects (SCEs) of multi-gate transistors, Dianlei et al. investigated the p-FinFETs with a CVD-grown GeSn channel [93]. For GeSn p-FinFETs grown on GeSnOI substrates with 8% Sn incorporation (Figure 40), compressive strain and hole mobility were found to be −0.9% and 208 cm^2^/V·s, respectively. Record low SS of 79 mV/decade for GeSn p-FETs were also achieved.

**Figure 41:**
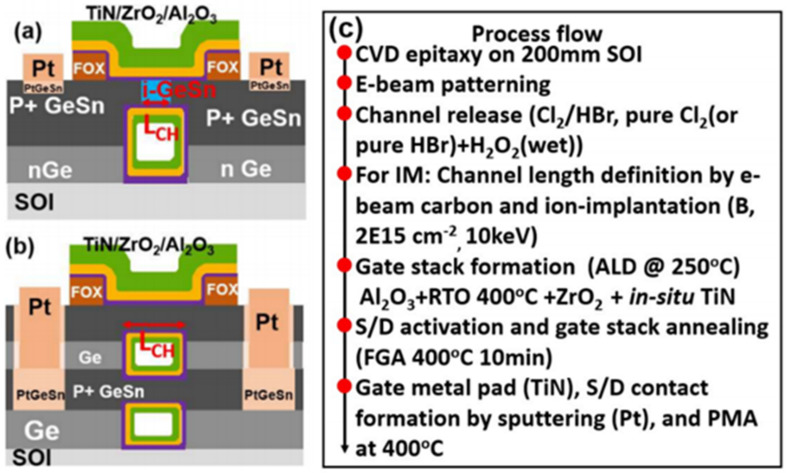
Schematic cross-sectional view of (**a**) single GeSn channel, (**b**) stacked GeSn nanowire pGAAFETs, and (**c**) process flow for devices. Reproduced with permission from [214], IEEE, 2017.

**Figure 42:**
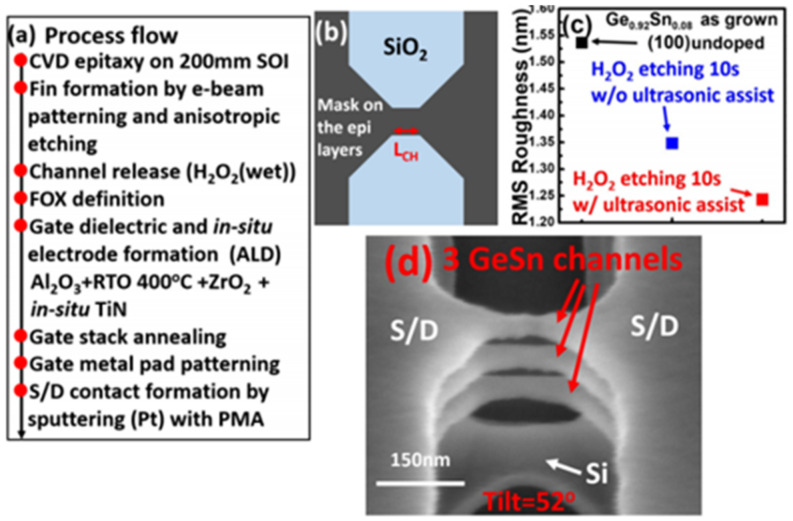
(**a**) Process flow for vertically stacked 3-GeSn nanosheet pGAAFETs; (**b**) top view after the fin formation; (**c**) RMS value for as-grown GeSn; (**d**) SEM image of stacked 3-GeSn nanosheets. Reproduced with permission from [91], IEEE, 2018.

Compared with FinFETs, gate-all-around (GAA) FETs hold better electrostatic control, which can reduce the SCEs for the gate-length scaling. With down-scaling came the proposition of a vertically stacked Si channel for GAAFETs in order to improve drive current [218,219]. Yu-Shiang Huang et al. systematically investigated the strain response, LF noise, and temperature-dependence properties of vertically stacked GeSn nanowire pGAAFETs [214] (Figure 41). Their experimental results showed that: (I) I_on_ = 1850 μA/μm was improved with higher Sn incorporation; (II) the 6.3% extra enhancement of I_on_ was observed due to the uniaxial compressive strain that occurred when using wafer bending; and (III) the SS for one-nanowire and stacked two-nanowire GAAFETs were 84 and 88 mV/dec, respectively. To further improve the drive current for GAAFETs at a given footprint (Figure 42), vertically stacked 3-GeSn nanosheet pGAAFETs were studied and the I_on_ was increased 1975 μA/μm at V_DS_ = −1 V.

**Figure 43:**
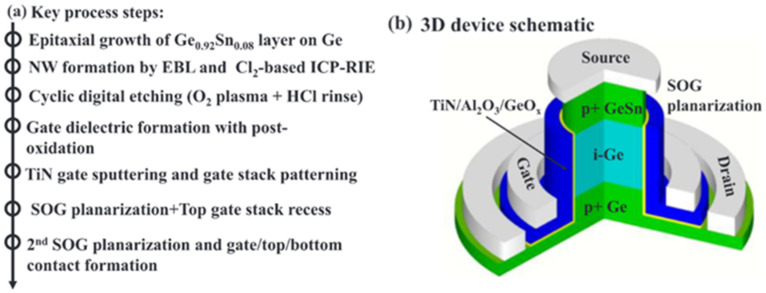
(**a**) Fabrication process and (**b**) 3D schematic of single vertical heterojunction GeSn/Ge GAA nanowire pMOSFETs. Reproduced with permission from [220], Elsevier, 2020.

Furthermore, a top–down approach was utilized to fabricate vertical heterojunction GeSn/Ge GAA nanowire pMOSFETs (Figure 43); with proper optimization, a record high I_on_/I_off_ (3 × 10^6^) was achieved [216].

Similar to the n–Ge material, n–GeSn suffers from a large resistance in metal-n–GeSn contacts mainly due to a strong Fermi pinning effect. To improve the performance of GeSn n-FETs, Yen Chuang et al. researched GeSn n-FinFETs and n-Channel MOSFETs: n^+^–GeSn contact; in situ doped n^+^–GeSn was grown by CVD, and Ni was employed as the contact metal [94]. With the increasing Sn content and n-type doping level, contact resistivity reduced to 3.8 × 10^−8^ Ω/cm^2^, which may be attributed to the bandgap shrinkage of GeSn (8% Sn incorporation). With the optimized n^+^–GeSn contact, the highest drive current and best SS for GeSn n-FinFETs were 108 A/m and 138 mV/dec, respectively (8% Sn incorporation) [91]. To suppress the dopant diffusion for S/D carrier activation, microwave annealing (MWA) was proposed. For GeSn with 4.5% Sn incorporation, GeSn nMOSFETs were found to possess an electron mobility of 440 cm^2^/V·s, suggesting that CVD-grown GeSn and MWA technologies are very promising for GeSn CMOS applications. For higher electron mobility, a 0.46% tensile strain was introduced to Ge_0.96_Sn_0.04_; due to the introducing of tensile strain, the carrier population in the Γ valley was higher. Thus, the electron mobility of GeSn nMOSFETs was further improved to 698 cm^2^/V·s [215].

This discussion shows that pTFETs, pFin-FETs, pMOSFETs, nMOSFETs, and vertically stacked nanowire pGAAFETs with CVD-grown GeSn layers have been extensively studied; breaking the bottleneck the n-doped or p-doped GeSn CVD growth technology is one of the main routes forward for high-performance GeSn transistors. Uniformly stacked nanowires or nanosheets with low surface roughness are of great importance for 5 nm CMOS technology nodes and beyond. More importantly, It should be noted that Henry. H. Radamson et al. explored Ni–(GeSn)_x_ contact formation [220]; the strain dependence, phase formation, and thermal stability of Ni–(GeSn)_x_ were systematically investigated, and they found that an Sn-rich surface impeded the diffusion of Ni, thus paving the way for the optimization of high-performance nanowire pGAAFETs.

## 6. Conclusions and Outlooks

In summary, the challenges and progress of GeSn CVD growth technology (including in situ doping technology and ohmic contact formation), GeSn lasers, GeSn detectors, and GeSn transistors were reviewed. Due to growth difficulties, such as the large lattice mismatch between GeSn and Si, the low solubility between Ge and Sn, and phase changes for Sn, more effort must be made in improving the quality of high-Sn-content GeSn materials, GeSn/SiGeSn heterostructures, and GeSn/SiGeSn QWs for high-performance electronic and optoelectronic devices, especially GeSn lasers and GeSn TFETs. Sn distribution uniformity and sharp GeSn/SiGeSn interfaces are the key issues in the development of room temperature, CW electrically pumped GeSn lasers. In addition, research on novel Si-based group IV materials, such as CSiGeSn and CSiGe [221,222,223], may pave the way for better strain compensation and lattice-mismatched laser structures.

## Figures and Tables

**Table 1:** Summary of reported B, BF_2_^+^, and P-doped GeSn via ion implantation in terms of year, institution, Sn content, doping type, doping concentration, activation temperature, and contact metal.

Year	Institution	Sn Content (%)	N-Type	P-Type	Doping Concentration (cm^−3^)	Activation Temperature (°C)	Contact Metal	Ref.
**2011**	Nagoya University	2–13	——	√	B: 8 × 10^19^	350–550	Ni	[163]
**2011**	CAS-IOS	3	——	√	BF_2_^+^: ——	400	Al	[164]
**2012**	NUS and CAS-IOS	2.4	√	——	P: 2.1 × 10^19^	400	Al	[165]
**2012**	NUS and CAS-IOS	4.2	√	√	P: 1 × 10^21^ BF_2_^+^: ——	400	Ni	[166]
**2012**	NUS and CAS-IOS	3–5.3	——	√	BF_2_^+^: >1 × 10^20^	300–500	——	[167]
**2013**	NUS	2.4	√	——	P: 2.1 × 10^21^	400	Al	[168]
**2013**	NUS	4.2	√	√	P: >1 × 10^20^ BF_2_^+^: >1 × 10^20^	400	Ni	[169]
**2013**	NUS	4.2	√	——	P: ——	450	Ni	[170]
**2013**	NUS	4.2	√	——	P: ——	400	Ni	[171]
**2013**	NUS and CAS-IOS	5.3	——	√	BF_2_^+^: ——	350	Ni and Ni–Pt	[172]
**2013**	NUS and CAS-IOS	4.1	——	√	BF_2_^+^: ——	——	——	[173]
**2013**	Stanford University	7	√	√	P: —— BF_2_^+^: ——	400	Ti/Ni	[174]
**2014**	NUS and AM	2.4	√	——	Hot P^+^: >1 × 10^20^	450	Ti/Ni	[175]
**2014**	NUS and AM	2.6	√	——	P: >1 × 10^20^	——	——	[176]
**2015**	CAS-IOS	3.2	√	——	P: 7.64 × 10^20^	500	Ni/Al	[177]
**2016**	Xidian University	4	——	√	BF_2_^+^: ——	——	Ni	[178]
**2016**	CAS-IOS	8	——	√	B: ——	300	Ni/Al	[179]
**2017**	Xidian University	4	——	√	BF_2_^+^: ——	——	Ni	[180]
**2017**	National Taiwan University	8	√	——	P: ——	300–350	Ni	[181]
**2019**	CAS-IOS	6	——	√	BF_2_^+^: ——	450	Ni/Al	[182]
**2020**	CAS-IOS	9	√	√	B: —— P: ——	500	Ni Al/Ti/Au	[183]
**2020**	National Chiao Tung University	2.8	——	√	BF_2_^+^: ——	400	Al	[184]

**Table 4:** Summary of reported GeSn photoconductive detectors in terms of Sn content, GeSn thickness, device structure, wavelength cutoff, and responsivity.

Year	Sn Composition	GeSn Thickness	Structure	Cutoff	Responsivity	Ref.
**2012**	9%	13 or 20 nm	GeSn/Ge 3QWs	2200 nm	0.1 A/W at 5 V	[58]
**2014**	0.9%	327 nm	Bulk	1800 nm	——	[59]
	3.2%	76 nm		1900 nm	——	
	7.0%	240 nm		2100 nm	0.18 A/W at 10 V	
**2014**	10%	95 nm	Bulk	2400 nm	1.63 A/W at 50 V	[60]
**2015**	10%	95 nm	Bulk	2400 nm	0.26 A/W	[61]
**2019**	12.5%	140 and 660 nm	Bulk	2950 nm	2 A/W	[63]
	15.9%	250 and 670 nm		3200 nm	0.044 A/W	
	15.7%	165, 585, and 254 nm		3400 nm	0.0072 A/W	
	17.9%	310, 550, and 260 nm		3350 nm	0.0038 A/W	
	20%	450 and 950 nm		3650 nm	0.0067 A/W	
	22.3%	380 and 830 nm		3650 nm	0.0032 A/W	

**Table 5:** Summary of reported GeSn PIN detectors in terms of Sn content, GeSn thickness, device structure, wavelength cutoff, and responsivity.

Year	Sn Composition	GeSn Thickness	Structure	Cutoff	Responsivity	Ref
**2009**	2%	350 nm	n–GeSn/i–GeSn/P-Si	1750 nm	-	[57]
**2016**	7%	200 nm	p–Ge/i–GeSn/n–Ge	2200 nm	0.15 A/W at 1 V	[62]
	10%	200 nm		2600 nm	0.07 A/W at 1 V	
**2018**	11%	700 nm	p–Ge/p–GeSn/i–GeSn/n–GeSn/n–Ge	2650 nm	0.32 A/W	[65]
**2019**	8%	25 nm	p^+^–Ge/i-QWs/n^+^–Ge	2000 nm	0.2 A/W	[66]

**Table 6:** Summary of the reported optically pumped FP cavity GeSn lasers in terms of structure, Sn content, thickness, cavity width, pumping laser, maximum operation temperature (T_max_), and threshold.

Year	Structure	Sn (%)	Thickness (nm)	Cavity Width (μm)	Pumping	T_max_ (K)	Threshold (kW/cm^2^)	Ref
**2015**	Bulk	12.6	560	5	Pulsed 1064 nm	90	1000 at 90 K 325 at 20 K	[70]
**2016**	Hetero	11	260 and 760	5	Pulsed 1064 nm	110	68 at 10 K 166 at 90 K 398 at 110 K	[71]
**2017**	Bulk	7.3	210 and 680	5	Pulsed 1064 nm	77	300 at 77 K	[73]
		9.9	280 and 850			140	117 at 77 K	
		11.4	180 and 660			140	160 at 77 K	
		14.4	250 and 670			160	138 at 77 K	
		15.9	210 and 450			77	267 at 77 K	
		16.6	160, 680, and 290			140	150 at 77 K	
		17.8	310, 550, and 260			180	171 at 77 K	
**2018**	Bulk	22.3	380 and 830	5	Pulsed 1064 nm	150	203 at 77 K 609 at 150 K	[75]
					Pulsed 1950 nm	180	137 at 77 K	
**2018**	QWs	13.8	22 (4×)	——	Pulsed 1950 nm	20	——	[83]
		14.4	31 (4×)	——		90	25 at 10 K 480 at 90 K	
**2019**	Bulk	20	450 and 970	5	Pulsed 1064 nm	120	516 at 77 K	[76]
				20		260	384 at 77 K	
				100		260	356 at 77 K	
				planar		260	330 at 77 K	
				5	Pulsed 1950 nm	140	132 at 77 K	
				20		270	88 at 77 K	
				100		270	47 at 77 K	
				planar		270	74 at 77 K	

**Table 7:** Summary of the reported optically pumped WGM cavity GeSn lasers in terms of structure, Sn content, thickness, disk size, pumping laser, maximum operation temperature (T_max_), and threshold.

Year	Structure	Sn (%)	Thickness (nm)	Disk Size (μm)	Pumping	T_max_ (K)	Threshold (kW/cm^2^)	Ref
**2016**	Bulk	8.5	800	8	Pulsed 1064 nm	90	125 at 50 K	[74]
		12.5	560	8		130	220 at 50 K	
**2018**	Hetero	16	418	20	Pulsed 1064 nm	230	134 at 15 K	[78]
							375 at 135 K	
							640 at 190 K	
							790 at 230 K	
**2018**	Hetero	14.5	380	8	Pulsed 1064 nm	100	300 ± 25 at 20 K 250 at 50 K	[84,86]
					Pulsed 1550 nm	120	420 ± 10 at 20 K	
	MQW-A	13.3	22 (10×)		Pulsed 1064 nm	100	35 ± 4 at 20 K	
					Pulsed 1550 nm	120	45 ± 3 at 20 K	
	MQW-B	13.5	12 (10×)		Pulsed 1064 nm	No lasing		
					Pulsed 1550 nm	20	——	
**2020**	Bonded bulk	5.4	40	9	Pulsed 1064 nm	85	0.8 at 25 K	[80]
				12		100		
				9	**CW 1550 nm**	72	1.1 at 25 K	
**2020**	Low TDD bulk	7	500	7	**CW 1550 nm**	80–95	10	[81,82]
		8.1		6			8	
		10.5		5			8.9 at 25 K	
				8			11.6 at 25 K	

**Table 8:** Summary of reported transistors with GeSn layers grown by CVD technology in terms of institution, transistor type, Sn content, subthreshold swing (SS), I_on_/I_off_ ratio, and V_DS_.

Year	Institution	Transistor Type	Sn Composition (%)	SS (mV/dec)	I_on_/I_off_	V_DS_ (V)	Refs
**2017**	University of Notre Dame	Ge/GeSn p-type TFETs	11 and 12.5	215	9.2 × 10^3^	−0.5	[99]
**2017**	NUS	GeSn FinFET on GeSnOI	8	79	>10^4^	−0.5	[93]
**2017**	National Taiwan University	Vertically Stacked GeSn Nanowire pGAAFETs	6 and 10	84	-	−1	[214]
**2017**	National Taiwan University	GeSn N-FinFETs	8	138	10^3^	-	[94]
**2018**	National Taiwan University	GeSn N-Channel MOSFETs	4.5	180	-	-	[215]
**2018**	National Taiwan University	Vertically Stacked 3-GeSn- Nanosheet pGAAFETs	7	108	5 × 10^3^	−0.5	[91]
**2020**	PGI 9	Vertical heterojunction GeSn/Ge gate-all-around nanowire pMOSFETs	8	130	3 × 10^6^	−0.5	[216]

## Data Availability

The data presented in this study are available on request from the corresponding authors.
